# A Systematic Review and Energy-Centric Taxonomy of Jamming Attacks and Countermeasures in Wireless Sensor Networks

**DOI:** 10.3390/s26020579

**Published:** 2026-01-15

**Authors:** Carlos Herrera-Loera, Carolina Del-Valle-Soto, Leonardo J. Valdivia, Javier Vázquez-Castillo, Carlos Mex-Perera

**Affiliations:** 1 Facultad de Ingeniería, Universidad Panamericana, Álvaro del Portillo 49, Zapopan 45010, Mexico; aherreral@up.edu.mx (C.H.-L.); lvaldivia@up.edu.mx (L.J.V.); 2 Department of Informatics and Networking, Universidad Autónoma del Estado de Quintana Roo, Chetumal 77019, Mexico; jvazquez@uqroo.edu.mx; 3 College of Engineering Technology, Rochester Institute of Technology, Rochester, NY 14623, USA; jmpiee@rit.edu

**Keywords:** energy-aware security, jamming attacks, energy harvesting, systematic literature review

## Abstract

Wireless Sensor Networks (WSNs) operate under strict energy constraints and are therefore highly vulnerable to radio interference, particularly jamming attacks that directly affect communication availability and network lifetime. Although jamming and anti-jamming mechanisms have been extensively studied, energy is frequently treated as a secondary metric, and analyses are often conducted in partial isolation from system assumptions, protocol behavior, and deployment context. This fragmentation limits the interpretability and comparability of reported results. This article presents a systematic literature review (SLR) covering the period from 2004 to 2024, with a specific focus on energy-aware jamming and mitigation strategies in IEEE 802.15.4-based WSNs. To ensure transparency and reproducibility, the literature selection and refinement process is formalized through a mathematical search-and-filtering model. From an initial corpus of 482 publications retrieved from Scopus, 62 peer-reviewed studies were selected and analyzed across multiple dimensions, including jamming modality, affected protocol layers, energy consumption patterns, evaluation assumptions, and deployment scenarios. The review reveals consistent energy trends among constant, random, and reactive jamming strategies, as well as significant variability in the energy overhead introduced by defensive mechanisms at the physical (PHY), Medium Access Control (MAC), and network layers. It further identifies persistent methodological challenges, such as heterogeneous energy metrics, incomplete characterization of jamming intensity, and the limited use of real-hardware testbeds. To address these gaps, the paper introduces an energy-centric taxonomy that explicitly accounts for attacker–defender energy asymmetry, cross-layer interactions, and recurring experimental assumptions, and proposes a minimal set of standardized energy-related performance metrics suitable for IEEE 802.15.4 environments. By synthesizing energy behaviors, trade-offs, and application-specific implications, this review provides a structured foundation for the design and evaluation of resilient, energy-proportional WSNs operating under availability-oriented adversarial interference.

## 1. Introduction

WSNs have emerged as a critical technology for numerous applications, including environmental monitoring, healthcare, industrial automation, and military surveillance [1,2,3]. These networks consist of small, energy-constrained sensor nodes that communicate wirelessly to collect and transmit data. However, their reliance on open wireless communication makes them highly susceptible to security threats [4], among which jamming attacks pose a particularly severe challenge [5]. Jamming disrupts network operations by intentionally emitting interfering signals to degrade or completely block legitimate communications. Given that WSNs often operate in hostile environments, understanding and mitigating jamming threats is paramount to ensuring their reliability and robustness [6].

WSNs are vulnerable to a wide array of security threats that can compromise their availability, integrity, and confidentiality. We are witnessing escalating congestion within the wireless communication spectrum [7]. This intensification of network traffic poses significant challenges for data transmission, connectivity, and overall system performance in densely populated wireless environments. This expansion has introduced various challenges related to system reliability, data privacy, and security vulnerabilities [8]. Some of the most common attacks include eavesdropping, in which an unauthorized entity secretly listens to network transmissions to extract sensitive information, and Sybil attacks, where a malicious node forges multiple identities, leading to routing inefficiencies and security breaches. Other major threats include wormhole attacks, which allow an adversary to tunnel packets from one location to another, bypassing normal routing mechanisms, and blackhole attacks, in which a compromised node falsely advertises optimal paths to attract traffic and then drops or manipulates received packets. Similarly, sinkhole attacks lure network traffic towards a malicious node, disrupting the normal data flow. Denial-of-service (DoS) attacks further degrade network performance by exhausting network resources and preventing legitimate nodes from functioning correctly. Among these security threats, jamming attacks are particularly devastating as they directly affect the PHY and MAC layers, rendering communication unreliable or even impossible.

Jamming attacks in WSNs can be categorized into different types based on their transmission patterns, power levels, and attack strategies. One of the most basic forms is constant jamming, where the attacker continuously emits high-power noise or random signals. Jamming attacks refer to the deliberate generation of electromagnetic interference that undermines the integrity and security of networks, particularly at the perception layer. This type of interference can disrupt communication signals, effectively compromising the performance and reliability of network systems [9]. While simple in execution, this type of jamming is highly energy-consuming for the attacker. A more deceptive approach involves transmitting seemingly legitimate packets, tricking sensor nodes into staying in a continuous receiving state, thereby disrupting normal communication without requiring brute-force interference. Another significant jamming strategy is random jamming, where the attacker alternates between transmitting jamming signals and remaining silent. This technique conserves energy while still effectively disrupting network performance. However, the most sophisticated and difficult-to-detect form of jamming is reactive jamming, in which the attacker remains idle and only transmits interference when it detects legitimate communication [10]. This strategy is particularly challenging to counteract because it minimizes unnecessary power consumption while remaining stealthy. Wireless sensor networks are particularly vulnerable to jamming attacks because their operation is tightly constrained by energy availability, lightweight hardware, and low-power radio interfaces. These constraints limit both the complexity of defensive mechanisms and the ability of sensor nodes to sustain prolonged countermeasures under interference. From the attacker’s perspective, jamming strategies exhibit markedly different energy profiles. Continuous interference patterns, such as constant or deceptive jamming, tend to be more easily detectable but impose a high energy burden on the jammer itself. In contrast, intermittent strategies, including random and reactive jamming, exploit protocol timing and channel activity to achieve disruption with significantly lower energy expenditure, making them more difficult to counter effectively.

**Figure 1:**
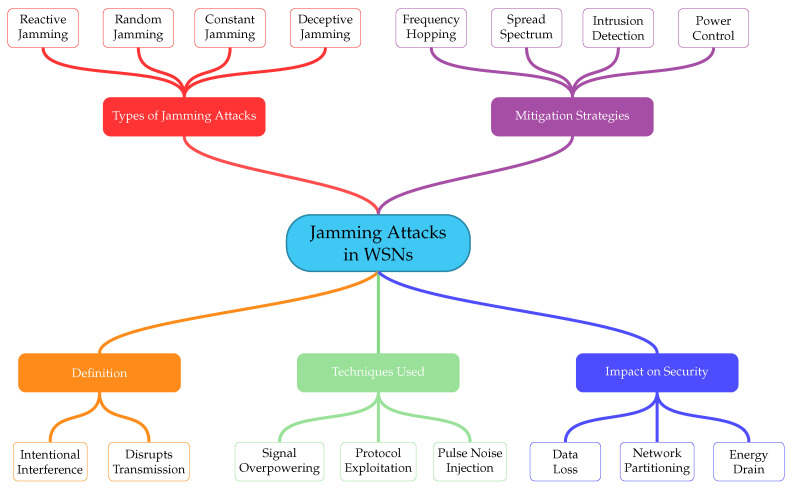
Jamming attacks in WSNs.

Conventional anti-jamming techniques, such as frequency hopping or spread-spectrum approaches [11], were originally developed for more capable wireless systems and are often impractical in WSN deployments due to hardware simplicity and stringent power budgets. As a result, recent research has shifted toward mitigation strategies that are explicitly tailored to resource-constrained environments. These include machine learning-based detection methods [12,13], adaptive transmission and duty-cycling schemes, and energy-aware countermeasures designed to minimize defensive overhead. In parallel, cross-layer defense approaches have gained attention by coordinating physical, MAC, and network-layer mechanisms, allowing WSNs to respond to jamming in a more flexible and energy-proportional manner. Figure 1 provides a conceptual overview of jamming attacks in wireless sensor networks, including their main types, techniques, impacts, and representative mitigation strategies.

### 1.1. Contributions

The main contributions of this work are summarized as follows:

- We conduct a systematic literature review covering publications from 2004 to 2024 that analyze jamming and anti-jamming mechanisms in wireless sensor networks from an explicit energy-centric perspective.
- We formalize the literature search and refinement process through a mathematical optimization model, enhancing transparency, reproducibility, and methodological rigor beyond conventional SLR practices.
- We adopt an energy-centric classification framework that organizes jamming strategies and countermeasures according to their energy behavior, attacker–defender energy asymmetry, and long-term sustainability implications, rather than solely by protocol layer or attack modality.
- We synthesize and systematize the energy-related performance metrics and experimental assumptions most commonly reported in IEEE 802.15.4-based jamming studies, providing a unified interpretative context for heterogeneous results.
- We identify cross-layer energy patterns, trade-offs, and methodological gaps that limit comparability and reproducibility, and we outline prioritized research directions for energy-aware and resilient WSN design.

### 1.2. Motivation

WSNs play a foundational role in contemporary smart environments, ranging from critical infrastructure monitoring to pervasive healthcare and environmental surveillance. However, their intrinsic energy limitations and reliance on open wireless media render them exceptionally vulnerable to jamming attacks. Such deliberate interference can severely degrade communication efficiency, accelerate energy depletion, and fragment network connectivity. Despite the increasing volume of publications over the last two decades addressing jamming mitigation, the intersection between jamming impact and energy consumption remains underexplored. This is particularly true when considering both the jammer’s energy expenditure and the response mechanisms of the sensor network.

This review is motivated by the urgent need to systematize and evaluate the state of the art in energy-aware jamming and anti-jamming strategies. From a scientometric perspective, our analysis spans two decades (2004–2024), drawing upon a rigorously curated corpus of 62 publications filtered from over 480 initial entries indexed in Scopus. The upward trajectory in both publication count and citation frequency since 2015 signals an intensifying scholarly and industrial focus on the energy implications of jamming. Keyword co-occurrence, citation clustering, and technological mapping further reveal a growing interdisciplinarity, with strong linkages to energy-efficient computing, secure communications, and machine learning (ML) in embedded systems.

This article contributes a novel angle to the state of the art by:

- Introducing an energy-centric taxonomy of jamming strategies, highlighting techniques such as reactive, deceptive, and cyclic prefix jamming that aim to optimize interference impact per joule consumed;
- Critically analyzing energy dissipation patterns in transmitters and receivers during attack scenarios, an aspect that has been scarcely addressed in previous reviews;
- Bridging theoretical frameworks with empirical findings through energy consumption models and duty-cycle metrics reported in the literature, including available real-world testbed evidence;
- Identifying cross-layer defense strategies that integrate energy-aware scheduling, adaptive power control, and lightweight detection algorithms.

By focusing explicitly on the energy dimension of jamming in WSNs, this review not only fills a significant knowledge gap but also provides actionable insights for designing next-generation WSN architectures that are both resilient and sustainable. In doing so, it supports the long-term deployment of secure, efficient, and intelligent sensor-based systems in critical application domains.

Despite the extensive literature on jamming attacks and countermeasures in wireless sensor networks, existing surveys rarely examine these threats from an explicit energy-centric perspective. Most prior studies either focus on the functional impact of jamming or assess defensive mechanisms without quantifying their energy overhead, resulting in fragmented evaluations, heterogeneous metrics, and limited cross-layer integration. This gap is particularly critical as modern WSNs increasingly rely on ultra–low-power architectures, duty-cycled operation, and energy-harvesting mechanisms. Addressing this deficiency, the present work pursues three core objectives: (i) to systematically synthesize two decades of research on jamming and anti-jamming techniques from an energy-aware standpoint; (ii) to formalize the literature-selection process through a rigorous mathematical search-and-refinement model that enhances transparency and reproducibility; and (iii) to evaluate and compare the energy implications of offensive and defensive strategies across physical, MAC, and network layers. Accordingly, this review proposes an energy-centric taxonomy of jamming attacks and countermeasures, constructs unified comparative tables of energy metrics and methodological characteristics, and identifies persistent gaps and emerging research challenges relevant for the design of sustainable, resilient, and energy-efficient WSN architectures.

The remainder of this article is organized as follows. Section 2 presents the related work and introduces a comprehensive thematic framework on energy-oriented jamming in WSNs. Section 3 describes the systematic literature review methodology and the mathematical model used to formalize the search and refinement process. Section 4 reports the main findings derived from the selected studies, highlighting quantitative trends and energy-related dynamics. Section 5 provides a critical discussion of these results and their implications for the design of resilient and energy-efficient WSN architectures. Finally, Section 6 concludes the study and outlines directions for future research.

### 1.3. System Model and Scope

This review considers WSNs compliant with the IEEE 802.15.4 standard [14], which provides the technological baseline for the majority of energy-aware jamming and anti-jamming studies analyzed between 2004 and 2024. The typical system model consists of battery-powered sensor nodes communicating with a coordinator or sink node using low-rate, low-power wireless links. Network topologies reported in the reviewed literature include star-based configurations, commonly used in monitoring and control applications, as well as multi-hop tree or mesh structures adopted in larger-scale deployments.

From a protocol perspective, this review focuses on the PHY and MAC layers defined by IEEE 802.15.4, which govern channel access, carrier sensing, retransmissions, and radio duty cycling. Together, these mechanisms strongly influence jamming effectiveness and energy consumption. Higher-layer protocols (e.g., routing or application logic) are considered insofar as they interact with PHY/MAC behaviors and contribute to cross-layer energy effects under interference.

Beyond enumerating the operating bands and deployment scenarios, the scope definition was intentionally aligned with the intrinsic architectural constraints imposed by IEEE 802.15.4, as these constraints fundamentally shape both jamming effectiveness and energy consumption patterns. In particular, features such as low-duty-cycle operation, carrier-sense multiple access with collision avoidance (CSMA/CA), narrowband channels, and strict radio-on-time minimization introduce protocol-level regularities that are repeatedly exploited by energy-efficient jammers. These characteristics differentiate IEEE 802.15.4-based WSNs from higher-throughput or always-on wireless systems and explain why jamming strategies that are marginally effective elsewhere can become disproportionately energy-draining in this context. By explicitly anchoring the review to these standard-compliance assumptions, the manuscript frames jamming not as a generic interference problem, but as a protocol-aware, energy-asymmetric availability attack. This clarification is essential to interpret the reviewed results coherently and to explain why energy-centric taxonomies and cross-layer analyses are necessary for IEEE 802.15.4 deployments, rather than optional refinements.

The reviewed studies primarily assume operation in unlicensed industrial, scientific, and medical (ISM) frequency bands supported by IEEE 802.15.4, including the 2.4 GHz ISM band and the sub-GHz 868/915 MHz bands. These bands exhibit markedly different propagation characteristics, interference environments, and energy trade-offs. Deployments in the 2.4 GHz band typically experience higher levels of external interference due to coexistence with Wi-Fi, Bluetooth, and other technologies, which can exacerbate both accidental and intentional jamming and increase retransmission-related energy costs. Such coexistence dynamics and channel access contention in unlicensed bands have been explicitly modeled in prior analytical studies [15,16]. In contrast, sub-GHz deployments benefit from improved propagation and penetration, often enabling longer communication ranges and lower transmit power, but remain vulnerable to wideband or high-power jamming strategies that can affect multiple channels simultaneously.

For clarity and to delimit the analytical scope of this review, 3GPP-standardized IoT systems, including NB-IoT, LTE-M, and NR/NR-U, are explicitly excluded. This exclusion is intentional and methodologically motivated. IEEE 802.15.4-based wireless sensor networks fundamentally differ from 3GPP IoT technologies in their physical-layer design, spectrum access mechanisms, scheduling models, and energy management strategies. IEEE 802.15.4 deployments rely on contention-based medium access in unlicensed bands, aggressive duty cycling, and ultra-low-power radio operation. These characteristics directly couple interference, retransmissions, and radio-on time to node energy depletion. In contrast, 3GPP IoT systems operate under centrally scheduled access, cellular-grade synchronization, and controlled power management, which lead to qualitatively different jamming dynamics and attacker–defender energy interactions. As a consequence, the energy-centric behaviors observed under jamming in IEEE 802.15.4 networks, such as disproportionate energy drain induced by reactive or protocol-aware interference, cannot be directly extrapolated to 3GPP-based IoT systems without redefining the threat model, evaluation metrics, and energy assumptions [17]. Explicitly excluding these technologies therefore preserves the internal consistency of the system model, ensures comparability across the reviewed studies, and prevents overgeneralization of energy-related conclusions beyond the architectural context in which they are valid.

Throughout this review, jamming is understood as any intentional interference strategy aimed at degrading network availability by disrupting packet reception, forcing repeated transmissions, or prolonging radio activity. From an energy-centric viewpoint, such attacks translate into energy-depletion mechanisms, where the attacker seeks to maximize the defender’s energy expenditure per successfully delivered bit. Accordingly, this work emphasizes how design choices specific to IEEE 802.15.4, including channel access modes, duty-cycling policies, and frequency band selection, shape the energy dynamics of jamming attacks and countermeasures.

Finally, the insights synthesized in this review are mapped to representative IEEE 802.15.4 deployment scenarios commonly discussed in the literature, including industrial monitoring and control systems, smart building and infrastructure sensing, and healthcare or wearable sensing applications. While the survey does not target a single application domain, this contextualization supports the interpretation of energy trade-offs and the practical relevance of the reviewed techniques across diverse real-world use cases.

Beyond serving as background context, coexistence and unlicensed-band access characteristics directly condition the energy efficiency and comparability of jamming evaluations in IEEE 802.15.4-based WSNs. Analytical and system-level studies have shown that channel occupancy, contention behavior, and access dynamics in unlicensed bands fundamentally shape packet loss, retransmission patterns, and radio-on time, thereby influencing both attacker effectiveness and defender energy depletion. Consequently, energy-centric interpretations of jamming attacks and countermeasure performance cannot be decoupled from the underlying coexistence assumptions inherent to unlicensed-band operation, as these assumptions materially affect attacker–defender energy asymmetry and the validity of cross-study comparisons.

### 1.4. Threat Model (CIA-Oriented)

To provide a clear and standardized security context for the analysis conducted in this review, we adopt the well-established Confidentiality–Integrity–Availability (CIA) model as a conceptual threat-modeling framework. Within IEEE 802.15.4-based wireless sensor networks, jamming attacks primarily target the Availability dimension, accordingly aiming to disrupt timely communication rather than to compromise data confidentiality or message integrity directly.

From an operational perspective, jamming degrades availability by preventing successful packet reception, inducing repeated retransmissions, prolonging channel sensing periods, or forcing nodes to remain in active radio states. When viewed through an energy-centric lens, these effects translate into energy-depletion denial-of-service mechanisms, where the attacker seeks to maximize the energy expenditure of legitimate nodes per successfully delivered bit or per unit of useful network throughput, a behavior that has been empirically observed in IEEE 802.15.4 networks under Wi-Fi interference and sustained channel disruption [18].

While Confidentiality and Integrity are not the primary objectives of jamming attacks, prolonged availability degradation can indirectly affect higher-level security functions, such as authentication exchanges, key updates, or network management operations. Nevertheless, this review deliberately centers on availability-oriented threats, as they dominate the energy dynamics observed in the surveyed literature and align with the primary design constraints of low-power IEEE 802.15.4 deployments.

Accordingly, the taxonomy and synthesis presented in this work classify jamming strategies and countermeasures based on how they primarily impact network availability and energy consumption across the physical and medium access control layers. Other threat categories defined in broader security models, such as confidentiality breaches or data manipulation attacks, are considered outside the primary scope of this survey and are discussed only to the extent they intersect with availability and energy consumption under interference.

## 2. Related Work

Jamming attacks have long been recognized as a major threat to the reliability and stability of wireless sensor networks [19]. Their impact depends strongly on the adopted attack strategy, with reactive jamming standing out as particularly effective due to its selective activation and reduced energy expenditure. Given the stringent resource constraints of WSNs, conventional anti-jamming techniques are often difficult to deploy in practice, motivating the exploration of energy-aware and adaptive countermeasures. As a result, recent research increasingly focuses on intelligent detection mechanisms and mitigation strategies that explicitly account for energy limitations in realistic deployments.

Sensor nodes are especially vulnerable to radio interference when adversaries exploit the operational characteristics of data link layer protocols. In this context, energy-efficient jamming has emerged as a critical concern, as it allows attackers to sustain interference while maintaining a low power footprint. Ensuring robustness at both the physical and link layers is therefore essential for preserving reliable data transmission under adversarial conditions [20].

A fundamental challenge in WSNs is the limited energy availability of sensor nodes, which stems from their compact hardware design and dependence on finite power sources. This limitation is compounded by the exposure of wireless communication to multiple security threats, including eavesdropping, traffic analysis, and data injection attacks, all of which can undermine network integrity and confidentiality [21]. While these threat categories provide general context, the present study is strictly focused on availability degradation caused by jamming and interference-based attacks, particularly from an energy-consumption perspective.

Among the different attack models, reactive jamming has been widely studied due to its ability to concentrate interference during active transmission periods. By targeting only legitimate communication events, this strategy maximizes disruption while minimizing the attacker’s energy consumption, making detection and mitigation particularly challenging in energy-constrained environments.

To provide a structured overview of how energy considerations intersect with security challenges in WSNs, this section synthesizes prior research using a thematic perspective. Rather than presenting isolated contributions, the literature is organized into key research domains that reflect recurring methodological choices, protocol layers, and energy-related objectives.

### 2.1. Comprehensive Thematic Framework of Energy-Oriented Jamming in WSNs

WSNs play a central role in applications such as smart environments, industrial automation, and the Internet of Things. However, their reliance on low-power devices and open wireless channels makes them particularly exposed to intentional interference, including jamming attacks. From an energy-oriented perspective, the study of jamming in WSNs reveals a diverse set of approaches aimed at characterizing attack behavior, evaluating defensive mechanisms, and quantifying their impact on energy consumption and network efficiency.

The reviewed literature spans multiple protocol layers and methodological viewpoints. Some works focus on modeling jamming strategies and their energy implications, while others propose detection, diagnosis, or mitigation techniques designed to limit energy depletion. Additional studies address energy modeling, optimization, and harvesting mechanisms, highlighting how interference affects different layers of the protocol stack and how countermeasures can be adapted accordingly. Finally, a subset of contributions adopts cross-layer or application-driven perspectives, reflecting the diversity of deployment scenarios in which energy-aware jamming must be addressed.

Following the methodology introduced in Section 1, the selected studies are grouped into eight thematic areas, summarized below, to facilitate comparison across approaches and to identify recurring trends and open challenges.

1. Energy-Oriented Jamming Attacks in WSNs.
2. Detection and Defense Strategies against Jamming.
3. Energy Consumption under Jamming.
4. Energy Harvesting Techniques for Resilience against Jamming.
5. Medium Access Protocols and Link Layer under Jamming.
6. Routing under Jamming Conditions.
7. Physical-Layer and Spread Spectrum Defenses against Jamming.
8. Applications and Use-Case Scenarios of Jamming in WSNs.

#### 2.1.1. Energy-Oriented Jamming Attacks in WSNs

In WSNs, jamming attacks pose a critical challenge not only because of their disruptive impact on communication, but also due to their pronounced energy implications. Unlike conventional cyber threats, jamming directly exploits the energy budget of sensor nodes by forcing them into prolonged transmission, reception, or channel sensing states. This attack model leverages a fundamental energy asymmetry between the adversary, which is typically equipped with a stable or abundant power source, and the sensor nodes, which rely on severely constrained batteries. Analyzing jamming from an energy-oriented perspective is therefore essential to understand its long-term effects on network lifetime, node availability, and operational reliability in resource-limited WSN deployments. The energy implications associated with the primary jamming techniques utilized in WSNs are outlined below.

- Deceptive Jamming: The primary factor contributing to significant performance degradation in WSNs is the incessant emission of noise packets. This not only escalates energy consumption but also reduces the network’s effective data transfer rate, as it leads to an increased incidence of packet collisions that obstruct the communication channel. Consequently, the jamming behavior characterized by the continuous and random transmission of data incurs substantial energy expenditures for the jammer itself.
- Random Jamming: This sophisticated form of jamming utilizes an energy-conserving strategy that distinguishes it from more traditional jamming mechanisms, which typically operate with continuous transmission regardless of energy status. The random jammer intermittently alternates between active jamming and a low-power sleep mode based on a predefined schedule. This transition to sleep mode is crucial for optimizing energy efficiency. Consequently, the power consumption dynamics in WSNs become inherently random and unpredictable, which complicates the overall assessment of the impact of jamming activities.In their analysis, Babar et al. (2013) in [22] observe that in environments featuring a limited number of malicious nodes, the energy expenditure of the WSN subjected to random jamming is reduced by 5% to 10% compared to scenarios utilizing deceptive jamming. This difference can be attributed to the nature of random jamming, which allows the malicious nodes to periodically enter an idle state. As a result, this reduces persistent interference and collision rates within the WSN, leading to enhanced energy efficiency, even though it may introduce some increase in latency.
- Constant Jamming: This type of jamming involves continuous and arbitrary data transmission by the attacker, resulting in frequent channel collisions within the WSN. Both the attacker and the WSN experience a lack of energy efficiency due to this incessant interference. The resultant collisions necessitate retransmissions, consequently amplifying energy consumption across network nodes. However, the performance degradation associated with constant jamming is comparatively less severe than that observed in other attack types, as the jamming occurs at regular intervals, allowing for brief periods of operational normalcy. From the perspective of the jammer, energy efficiency is not achieved due to the need for continuous transmission, albeit with intermittent gaps. This is in stark contrast to random jamming techniques, where the attacker employs strategies to conserve energy. In their research, López-Vilos et al. (2021) in [23] analyze the energy consumption patterns of a constant jammer relative to the transmitter nodes. Their findings indicate that the constant jammer exhibits a 40% greater energy consumption (291.60 J of total consumption) compared to the XBee S1-based nodes in the WSN. They compare this approach to the deployment of a reactive jammer, highlighting that a constant attacker can consume up to 118% more energy.
- Reactive Jamming: This type of jamming attack presents a significant threat in WSNs. This form of jamming is characterized by the generation of noise packets in response to the detection of channel activity. The immediate proliferation of these noise packets leads to substantial packet corruption and loss due to the resultant surge in collisions. Consequently, the network is compelled to initiate retransmissions for the affected packets, thereby escalating the overall energy consumption among WSN nodes. This disruptive mechanism poses challenges to network reliability and efficiency. The jammer is distinguished by its intelligent attacks, which are made possible by its energy-saving approach compared to jammers that transmit continuously. This type of jammer is only activated when it detects an event on the channel. Otherwise, it remains in idle mode (idle mode or quiet state). The inherent design of the reactive jammer, remaining in an idle state for the majority of its operational time, optimizes energy efficiency for the attacker. This capability extends the duration during which the attacker can execute offensive maneuvers when required.

#### 2.1.2. Detection and Defense Strategies Against Jamming

In the context of WSNs, jamming attacks represent a major threat to reliability and availability, often triggering defensive responses that themselves incur non-negligible energy costs. An energy-aware perspective on mitigation strategies emphasizes the need to balance protection effectiveness with the additional energy overhead introduced by defensive mechanisms, particularly in networks where battery replacement is impractical. By jointly considering the attacker’s energy expenditure and the defensive strategy’s energy footprint, this perspective enables a more realistic assessment of resilience and supports the design of sustainable countermeasures for resource-constrained WSN environments.

In the realm of attack mitigation strategies, commonly referred to as anti-jamming techniques, two primary challenges arise in implementing them within WSNs: the constraints imposed by the capacity of processing robust algorithms and the limited energy resources, and the necessity to effectively minimize the frequency of attacks. For example, the Uncoordinated Frequency Hopping (UFH) scheme proposed by Strasser et al. (2008) in [24] presents significant storage and processing overhead, which consequently influences energy consumption. Nonetheless, they demonstrate that the approach maintains a high level of robustness against uncoordinated jamming. In contrast, Misra et al. (2010) in [25] introduced a swarm intelligence algorithm known as the “Ant System,” which effectively minimizes energy consumption while ensuring network resilience, particularly during high-intensity attack scenarios. This approach leverages decentralized problem-solving techniques inspired by ant behavior, enhancing network functionality and robustness under duress.

In the realm of mitigation strategies, both adaptive and energy-aware approaches are significant. One noteworthy contribution comes from Romeo et al. (2019) in [26], who develop a model grounded in game theory to enhance privacy at the physical layer of Cognitive Wireless Sensor Networks (CWSNs). Their method involves the strategic introduction of artificial noise within the communication spectrum, serving to obfuscate genuine data transmissions. This technique effectively fortifies security measures against privacy threats, particularly eavesdropping attacks. In this study, the authors describe a network architecture where nodes partition their operational time into discrete intervals. During the latter segment of these intervals, termed “T,” the nodes function as jammers by emitting artificial noise packets. This strategy effectively obfuscates the legitimate signal from an eavesdropper’s perspective. The simulations reveal that nodes possess the capability to halt noise transmission, thereby conserving energy while maintaining network functionality, thereby optimizing the trade-off between energy consumption and security. The findings indicate that implementing this security strategy results in a notable increase in energy consumption, specifically a 22% increase for nodes with fully charged batteries. However, this is mitigated by enabling nodes with depleting power reserves to prioritize their autonomy, thereby ensuring the long-term sustainability of the network. This adaptive approach enables the preservation of critical security measures without compromising operational capabilities, even in low-energy conditions.

López-Vilos et al. (2023) in [27] introduce a novel approach termed FCPA (Fairness Cooperation with Power Allocation), aimed at enhancing resilience against interference attacks through an energy-efficient, cluster-based self-healing strategy for WSNs. A key energy metric addressed in their work is residual energy, defined as the remaining energy in the batteries of network nodes at the conclusion of an operational period or upon the depletion of the first node’s battery. In their analysis, the authors found an average residual energy of 63% across all evaluated scenarios. Furthermore, they observed that the Cluster Head (CH) node was the initial component to deplete its battery, highlighting effective energy utilization throughout the network.

##### Energy-Efficient Protocols Against Attacks

Energy-efficient jamming at the MAC layer represents a particularly critical threat to wireless sensor networks, as it exploits the very design principles intended to prolong network lifetime. By selectively targeting channel access mechanisms, scheduling policies, or listening periods, such attacks can disrupt communication while maintaining a low energy footprint on the attacker side. This asymmetry makes MAC-layer jamming especially challenging to detect and mitigate in energy-constrained environments.

Several studies have investigated how the intrinsic characteristics of widely adopted MAC protocols influence their resilience under such adversarial conditions. Early analyses demonstrated that protocol determinism plays a central role in vulnerability. Protocols with fixed schedules or predictable access patterns can be exploited through protocol-aware jamming strategies that synchronize interference with active communication intervals. In this context, simulation-based evaluations of S-MAC, LMAC, and B-MAC revealed that protocols incorporating structured time-slot allocation tend to exhibit different resilience profiles, with LMAC consistently showing improved robustness when compared to contention-based alternatives [21,28]. Subsequent refinements further indicated that introducing controlled randomness in slot timing and duration can significantly reduce susceptibility to energy-efficient jamming.

Complementary investigations extended this analysis to adaptive MAC protocols, highlighting the role of dynamic duty cycling. Comparative studies involving IEEE 802.15.4 MAC, T-MAC, and S-MAC under both normal and adversarial conditions showed that protocols capable of adjusting active periods in response to traffic conditions achieve superior energy efficiency and throughput resilience [29]. By minimizing idle listening and shortening unnecessary transmission windows, adaptive mechanisms reduce the exploitable surface for jamming-induced energy depletion.

Beyond traditional WSN deployments, similar challenges arise in low-power wide-area network contexts. Although LPWAN protocols such as LoRaWAN are engineered for long-range, low-energy operation, they remain vulnerable to energy depletion attacks that artificially increase transmission or reception activity. Recent work has addressed this issue by embedding lightweight detection mechanisms directly within sensor nodes, enabling early identification of anomalous energy consumption patterns with minimal overhead [30]. These approaches demonstrate that localized, energy-aware detection can effectively mitigate depletion-oriented attacks without imposing significant additional power costs.

Other protocol-level innovations focus on leveraging channel diversity and multichannel access to balance energy efficiency and resilience. Multichannel MAC designs dynamically adapt channel selection and receiver wake-up timing based on sensed conditions, achieving low duty cycles while limiting exposure to persistent interference [31]. At higher layers, routing-oriented solutions complement MAC-layer defenses by redistributing traffic away from jammed regions and optimizing energy usage under adversarial pressure. Hybrid routing strategies that combine clustering and interference awareness have been shown to significantly extend network lifetime compared to baseline protocols [32,33]. More recent evaluations across multiple clustering-based routing schemes further confirm that protocol selection strongly influences energy consumption and resilience when jamming and interference coexist [34].

Overall, the reviewed evidence indicates that energy-efficient protocol design alone does not guarantee resilience against jamming. Instead, robustness emerges from the interaction between protocol determinism, adaptivity, and cross-layer coordination, emphasizing the need for energy-aware designs that explicitly account for adversarial exploitation of MAC and routing behaviors.

Finally, low-duty-cycle operation emerges as a defining characteristic of energy-efficient WSN deployments in adversarial environments. While such designs minimize baseline energy consumption, they also incentivize attackers to adopt more sophisticated and adaptive interference strategies. In this context, recent work has demonstrated that machine learning can be leveraged not only for defense, but also to enhance the efficiency of jamming attacks.

A representative example is the LearJam strategy proposed by Yang et al. [35], which illustrates how attackers can exploit observed transmission regularities to concentrate interference on active communication intervals. By learning probabilistic transmission patterns and aligning jamming activity accordingly, such approaches achieve higher disruption rates while maintaining low attacker energy expenditure. Reported evaluations indicate substantial gains in attack effectiveness compared to uninformed jamming strategies, underscoring how low-duty-cycle operation, while energy-efficient for legitimate nodes, can also increase susceptibility to learning-based adversaries.

#### 2.1.3. Energy Consumption Under Jamming

This theme examines the energy impact associated with both jamming attacks and the mitigation strategies employed to counter them. From an energy-centric standpoint, this impact must be analyzed from two complementary perspectives, namely that of the attacker and that of the wireless sensor network. On the attacker side, energy management determines how long malicious activity can be sustained, particularly when stealth or persistence is required. On the network side, defensive responses, while aimed at preserving data integrity and availability, often introduce additional energy expenditure through retransmissions, sensing, or protocol reconfiguration. This dual perspective highlights a central challenge in energy-aware WSN design: achieving security and resilience without accelerating energy depletion and compromising long-term network operation.

Key factors that significantly affect the power consumption of WSNs, regardless of jamming type, include the following:

- Vulnerability: WSNs exhibit inherent vulnerabilities primarily due to their constrained hardware and software capabilities, which limit the implementation of complex algorithms essential for mitigating jamming attacks. Sensor nodes, being the most resource-constrained elements of the network, face significant challenges in processing power and energy efficiency. It’s crucial to recognize that a sensor node becomes nonfunctional when its energy source is depleted, emphasizing the importance of energy management in the design of resilient WSNs.
- Threat of energy-efficient link-layer jamming attacks: This form of attack represents a significant risk to WSNs. An energy-efficient jammer can disrupt network communication for extended durations due to its low energy consumption during the jamming process. The potential for energy-efficient link-layer DoS jamming attacks is particularly concerning, as these attacks can effectively inhibit communication within the network for prolonged periods. This capability underscores the serious nature of the threat posed by such jamming mechanisms.
- Prolonged attack durations coupled with minimal power requirements: Energy-efficient jamming attacks, such as Periodic Slot Based Jamming (PSJ), proposed by Mahmood et al. (2011) in [36], are characterized by being energy-efficient, achieving high censorship rates (i.e., avoiding packet delivery) while maintaining a long lifetime advantage over the sensor node. The proposed approach leverages statistical analysis of network traffic, employing techniques such as K-means clustering and Expectation Maximization (EM) to gauge the periodic behavior of the MAC protocol in use. This enables the attacker to strategically time their interference while optimizing power consumption by remaining dormant during non-active periods.
- Impact on Authentic Communication: Jamming significantly affects communication challenges in WSNs, including reduced link reliability, increased packet latencies, and interruptions in end-to-end routing. These issues can lead to adverse energy consumption impacts, potentially rendering a sensor node inoperable if its energy source is drained due to heightened energy demands, such as those arising from retransmission processes.

**Figure 2:**
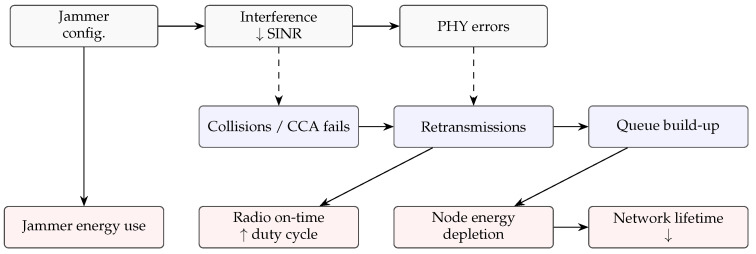
Energy impact flow diagram: from jammer configuration to PHY/MAC degradation and network-level energy impact.

Figure 2 summarizes the energy impact of jamming in WSNs, illustrating how interference at the PHY and MAC layers translates into increased radio activity, node energy depletion, and reduced network lifetime.

#### 2.1.4. Energy Harvesting Techniques for Resilience Against Jamming

In the context of WSNs, energy harvesting emerges as a key solution to address one of the main challenges: the need to replace or recharge the batteries of sensor nodes, especially in hard-to-reach environments or long-term applications. Unlike traditional networks that rely on limited power sources, WSNs can benefit from energy harvesting techniques to extend their autonomy and ensure continuous and sustainable operation. There are various energy sources that can be exploited by sensor nodes, among which the following stand out:

1. Solar energy, which is one of the most widely used sources due to its high availability and relatively stable power output in open environments;
2. Mechanical vibrations or kinetic energy, harvested through piezoelectric materials or electromagnetic generators, useful in industrial or urban environments with constant motion;
3. Thermal gradients, which allow energy to be obtained from temperature differences, for example, between the human body and the environment in biomedical applications;
4. Radio frequency (RF), which enables energy harvesting from electromagnetic waves present in the environment, such as those emitted by Wi-Fi networks, radio, or microwaves.

Each of these sources has its advantages and limitations in terms of energy density, availability, and conversion requirements, so the choice of the most suitable harvesting technique depends on the deployment environment and the energy consumption profile of the sensor node.

Tran et al. (2020) in [37] investigate the communication performance in energy harvesting WSNs, focusing specifically on scenarios involving coordinated attacks by an inhibitor and an eavesdropper. Their research presents a novel algorithm designed to optimize the timing of energy harvesting. The findings demonstrate that implementing these optimized policies leads to a reduction in energy consumption while simultaneously enhancing the overall quality of communication within the network. The study focuses particularly on RF energy harvesting from environmental sources, including mobile tower antennas, wireless local area network (WLAN) signals, or TV signals. Specifically, the protocol encourages sensors to harvest energy at the highest interference levels while simultaneously transmitting data packets through channels characterized by the lowest interference. This strategy enhances the reliability of communications by optimizing energy acquisition and minimizing the interference’s impact on data transmission.

Complementing this approach, Zhu et al. (2017) in [38] address several energy-related challenges faced by Industrial Wireless Sensor Networks (IWSNs). They emphasize the need for energy-efficient design strategies, the implementation of joint defenses against both interference and espionage, and the investigation of energy harvesting mechanisms. The authors classify energy harvesting into two primary approaches. The first involves harvesting ambient energy, such as the kinetic energy generated by machine vibrations, which could enable a continuous power supply to the transceiver if this energy source is consistently available. The second approach involves wireless energy transfer, either through electromagnetic induction or by transmitting microwave frequencies with the aid of rectennas. Using the latter principle, they propose an energy pattern-assisted simultaneous wireless information and energy transfer scheme, where information is embedded in energy patterns designed specifically for wireless energy transfer.

In alignment with these works, Do et al. (2019) in [39] investigate two energy harvesting methodologies to sustain the operation of sensor nodes: solar energy harvesting and radio frequency (RF) energy harvesting. Within the scope of their analysis, solar energy serves as the primary energy source, harnessed via solar cells at the source node. This node subsequently captures RF energy, predominantly from neighboring sensor nodes and the eavesdropper or intruder. The authors indicate that the efficiency of RF energy harvesting typically ranges between 50% and 75% for a power input within a 100-m range. They acknowledge that the quantity of solar energy harvested is significantly influenced by environmental factors, including cloud cover, surface dust accumulation on the solar cells, and variations in illumination. Their simulations reveal that the mean energy harvested (Eh,avg) is 10 packets per time interval, where one packet of energy equals 0.2 mJ. Therefore, on average, they collect 2 mJ of solar energy per time interval (10 packets × 0.2 mJ/packet). Additionally, they report that the power density of the RF energy harvester varies from $0.1\;\mu W/{\mathrm{cm}}^{2}$ to $1\;\mathrm{mW}/{\mathrm{cm}}^{2}$.

Expanding on the theme of integrating energy harvesting into system functionality, Aboulhassan and Abd El-Malek (2021) in [40] present a novel battery-free jamming localization technique designed for WSNs using battery-free RFID sensor tags that collect energy from the signal emitted by a powerful jammer. By quantifying the power received at these energy-harvesting nodes, the system can determine the distance to the jammer and subsequently estimate its precise location. The authors utilize Texas Instruments (TI) MSP430FR5994 microcontroller devices as sensor nodes. The experimental setup includes an evaluation board equipped with the Power Harvester P2110B chip, which is responsible for energy harvesting and produces an output voltage between 1.8 V and 5.25 V to transfer harvested RF energy to the TI nodes. In addition, a PowerCast transmitter is employed as the power source and acts as the jammer. The energy harvested by the P2110B chip is inversely related to the distance from the transmitter, with measurements indicating energy collection of 2.606 mJ at a distance of 1 m and 1.0749 mJ at 4 m. These results highlight that as the distance between the PowerCast transmitter (acting as the jammer) and the evaluation board increases, the time needed to collect energy also increases, and vice versa.

#### 2.1.5. Medium Access Protocols and Link Layer Under Jamming

The link layer is often the first line of defense against jamming because it governs how nodes access the communication channel. Protocols like CSMA, TDMA, and IEEE 802.15.4 are highly susceptible to manipulation by adversaries who exploit contention mechanisms or time-slot allocations. Research in this category examines both the vulnerabilities of these protocols and the proposed adaptations, such as randomized backoff, cooperative retransmission, or hybrid scheduling schemes. These innovations aim to preserve throughput and fairness while minimizing additional energy costs, reinforcing the robustness of the network at a foundational level. The study by Nguyen et al. (2019) in [41] addresses the problem of denial-of-service and jamming attacks in wireless sensor networks, proposing an anomaly detection system based on fuzzy logic as an alternative to traditional Shannon entropy methods. Experimental results show that, in a scenario with 84 inserted anomalous events, the classical entropy-based approach detected only 45 cases, whereas the fuzzy logic model identified 75 anomalous cases and 5 suspicious ones, significantly reducing the false positive rate that typically affects conventional statistical methods. This improvement in detection capability is crucial at the link layer, where attacks such as jamming exploit the weaknesses of medium access protocols (CSMA, TDMA, IEEE 802.15.4), undermining channel fairness and efficiency. In this regard, fuzzy logic not only provides a 66% increase in detection accuracy compared to classical techniques but also reduces computational complexity, thereby optimizing the energy consumption of resource-constrained nodes and strengthening network robustness in IoT scenarios. Kasireddy et al. (2014) in [42] analyze energy-efficient outsider attacks on IEEE 802.15.4 beacon-enabled networks, showing how the deterministic superframe structure and beacon interval can be exploited to perform selective jamming at the link layer. They describe three attacks—beacon jamming, active period jamming, and malicious PAN creation—all effective even when traffic is encrypted. Simulations using OMNeT++ and Castalia revealed severe impacts: Node 0 experienced 780 buffer overflows and remained associated for only 281 s, while Node 4 was disconnected in just 88 s, with only 7% association time. These results demonstrate that an attacker can outlast legitimate nodes by interfering only during critical intervals, thereby undermining fairness and efficiency of access protocols such as CSMA/CA in IEEE 802.15.4. The study concludes that countermeasures like randomizing beacon intervals and superframe durations are necessary to strengthen link-layer robustness against jamming. Osanaiye et al. (2022) in [43] evaluate the impact of constant jamming denial-of-service attacks on two reactive routing protocols widely used in WSNs: Ad hoc On-Demand Distance Vector (AODV) and Dynamic Source Routing (DSR). Using NS2 simulations in a clustered WSN topology, the authors measured performance metrics such as packet sending ratio (PSR), packet loss, and transmitted packets. Results revealed that cluster head nodes, which play a critical role in data aggregation, were the primary targets, with PSR values dropping to 0.62 for node 7 and 0.96 for node 3, while all other nodes maintained PSR≈1. Similarly, both cluster heads recorded the highest packet losses (27 packets each), demonstrating their vulnerability under targeted jamming. Importantly, the study found no significant difference in resilience between AODV and DSR, indicating that both protocols are equally exposed to constant jamming. These findings confirm that, despite differences in route discovery mechanisms, reactive routing protocols cannot mitigate link-layer jamming attacks, emphasizing the need for new defenses that reinforce fairness and efficiency at the medium access and routing layers. Cheng et al. (2019) in [44] expose critical vulnerabilities in WirelessHART networks, an IEEE 802.15.4-based industrial standard that relies on Time Slotted Channel Hopping (TSCH) and graph routing to ensure reliable, low-power communication in harsh industrial environments. Their study demonstrates that an attacker can silently eavesdrop on unencrypted Data-Link and Network Protocol headers to reconstruct routing paths, requiring as little as 1920 ms of snooping to derive a primary route in a 50-node WirelessHART testbed. Once routes and channel hopping sequences are cracked, selective jamming attacks can be launched by targeting only specific transmissions in designated time slots and channels. Compared to traditional jamming, this selective link-layer interference is highly energy-efficient and difficult to detect, undermining the fairness and robustness of medium access in TSCH-based protocols. The findings highlight that, despite their industrial adoption, WirelessHART and similar graph-routing schemes remain vulnerable at the link layer, and countermeasures such as encrypting headers or randomizing routing paths are essential to preserve reliability and security in critical infrastructure networks. Harbin and Mitchell (2011) in [45] investigate the impact of Sybil attacks in WSNs, particularly in scenarios that employ distributed beamforming as an energy-efficient alternative to multihop routing. In Sybil attacks, a single malicious node forges multiple identities, thereby compromising routing diversity and reducing the number of legitimate nodes participating in collaborative transmissions. Their analytic model shows that as the proportion of Sybil nodes increases, the signal-to-interference-plus-noise ratio (SINR) at the sink node significantly degrades, lowering the probability of successful beamforming reception. To mitigate this, the authors propose a reputation-based routing framework that integrates SINR feedback into a modified AODV protocol, enabling nodes to dynamically avoid Sybil-compromised clusters and route through more reliable supernodes. Simulation results across 30 topologies demonstrated that after approximately 300 route requests, the system converged to favor high-SINR supernodes, with 90% of traffic routed through clusters above the 4 dB decoding threshold. This approach reduces vulnerability at both the routing and physical layers, reinforcing fairness and reliability in medium access while avoiding the overhead of explicit Sybil detection algorithms.

#### 2.1.6. Routing Under Jamming Conditions

Routing in WSNs is particularly sensitive to jamming since multi-hop communication relies on the stability of intermediate nodes and links. Attacks that disrupt specific areas of the network can fragment connectivity, increase path lengths, and amplify energy consumption. This theme explores solutions such as energy-aware routing, clustering algorithms, and dynamic re-routing that adapt to interference in real time. By ensuring connectivity and balancing the load across the network, these strategies seek to extend the operational lifetime of WSNs even in environments where jamming is persistent or localized. In their article Tiloca et al. (2017) in [46], presented JAMMY as a distributed solution designed to maintain routing stability in WSNs under jamming conditions. The protocol dynamically modifies the assignment of TDMA slots, reducing the effectiveness of selective jamming to approximately 1/N, where N is the number of slots per superframe. Experiments conducted on the Indriya (TelosB) testbed with *N* = 30 showed that packet loss in jammed nodes decreased to around 4%, closely matching the theoretical value of 3.3%, demonstrating the system’s ability to preserve both connectivity and energy efficiency in environments affected by localized interference. Unlike centralized approaches, JAMMY introduces no additional communication overhead, as slot computation is performed locally, thus conserving bandwidth and node energy. Furthermore, it enables the integration of new nodes in fewer than five superframes, facilitating rapid adaptation to topological changes without compromising routing stability. Similarly, Zhu et al. (2012) in [47] extend the discussion by introducing an optimal adaptive antijamming framework for WSNs, which directly supports the goal of maintaining routing stability under jamming conditions. Their approach models the selection of anti-jamming techniques as a Markov Decision Process (MDP), enabling each node to dynamically adapt its defense strategy according to real-time channel conditions. The system integrates three primary countermeasures, namely transmission power adjustment, error-correcting codes (Reed–Solomon), and channel hopping, allowing nodes to minimize energy consumption while preserving packet delivery ratios (PDR). Simulation results using IEEE 802.15.4 parameters demonstrate that the adaptive policy improves PDR by more than 50% compared to static methods in areas within 8 m of the jammer, where baseline delivery fell below 50%. Additionally, the proposed method achieved 20% lower energy consumption than conventional channel-surfing techniques and maintained comparable latency (≈15 ms) while scaling efficiently up to 100 nodes without significant throughput degradation. Continuing the discussion on routing under jamming conditions, López-Vilos et al. (2023) in [27] proposed a clustering-based, energy-efficient self-healing strategy (FCPA: Fairness Cooperation with Power Allocation) that directly addresses the challenge of maintaining connectivity and route stability in WSNs exposed to jamming. Their method combines clustering, cooperative relaying, and adaptive power control to reroute data around jammed areas while balancing energy expenditure across the network. Using the log-distance channel model and a constant jammer emitting at 14 dBm, the authors simulate a 64-node IEEE 802.15.4 network under seven jammer locations. Results show that the FCPA algorithm sustains ≈ 63% residual energy while achieving nearly 1 Mb of transmitted data per scenario, outperforming classical approaches such as fixed-power (FP), power-allocation (PA), and cooperation-only (CP) schemes by over 50% in throughput. Notably, FCPA’s load-balancing mechanism ensures that the cluster-head nodes deplete first, a sign of optimized routing fairness and energy distribution. Compared to baseline methods that lose up to half the network’s coverage near the sink, the proposed strategy maintains full multi-hop connectivity, dynamically reconfiguring routes and cluster heads as jammer position changes.

#### 2.1.7. Physical-Layer and Spread Spectrum Defenses Against Jamming

At the physical layer, spread spectrum techniques like DSSS and FHSS, along with more advanced hybrid modulation schemes, have proven effective in reducing the impact of jamming. These methods disperse the signal over wider frequency ranges or hop across channels to evade interference. Although they offer strong resilience, their integration into WSNs must consider the energy cost of added complexity. Research in this theme focuses on striking a balance between robustness and efficiency, ensuring that physical-layer defenses not only secure communication but also align with the limited energy budgets of sensor nodes. In their study, Aasha Nandhini et al. (2012) in [48] propose a substantial enhancement to the conventional DSSS employed in the IEEE 802.15.4 standard. The approach consists of forming 3003 groups of five 15-chip PN sequences, selected randomly and in an uncorrelated manner, which expands the attacker’s search space and drastically reduces the probability of a successful jamming attack. In simulations conducted with 100 nodes deployed over a 500 × 500 m^2^ area, the traditional DSSS exhibited vulnerabilities, with a break probability of 0.36 after 500 attempts with a single jammer and up to 0.6 with 10 jammers in only 100 attempts; in contrast, the proposed scheme limited these probabilities to 0.16 and 0.28 under the same conditions. These findings indicate that, even under adverse scenarios with multiple attackers, the method preserves partial message integrity, as an adversary would only be able to recover isolated bits rather than the complete communication. In their study, Tsou and Chen (2018) in [49] introduce CCPT (Compression and Correctness-Preserving Top-k Query), a method that leverages compressive sensing (CS) combined with adaptive compressed data reduction (ACDR) and a link-neighborhood correctness verification scheme to improve query efficiency in WSNs. Using a dataset of 47,500 samples compressed into 1200 values, CCPT adaptively reduces the number of compressed values (Mmin) required for accurate Top-k recovery, achieving reliable reconstruction even when reducing data volume by up to 50% without loss of accuracy. Compared with existing methods such as IS (Impression Store), VQ (Verifiable Query), and VFTopk (Verifiable Fine-grained Top-k queries), the authors report that CCPT achieves equal recovery rates while requiring significantly fewer compressed values and substantially lower communication overhead. Experimental results on a Raspberry Pi 3 platform showed that CCPT reduced processing energy consumption to approximately 0.08 A, compared with up to 0.3 A for cryptography-based methods, and cut transmission energy by a factor of 1.5× to 2.2×. These results demonstrate that CCPT provides an effective balance between energy efficiency, data integrity, and query correctness, making it a practical approach for prolonging WSN lifetime under strict resource constraints. Hussain et al. (2022) in [50] present an innovative scheme for jammer localization in WSNs based on battery-free RFID nodes with energy-harvesting capabilities, which exploit the high power of the interfering signal to estimate the attacker’s position through path-loss models and triangulation. In a scenario with 30 dBm transmissions at 915 MHz, the authors conducted 10,000 simulations under log-normal shadowing conditions (σ between 0.5 and 3 dBm) and varying numbers of nodes (3–10), showing that the mean localization error decreases to ∼4.5 m with 10 active nodes and σ=1 dBm, while it can reach ∼10 m when σ=3 dBm. Experiments with real hardware using PowerCast P2110B harvesters (Powercast Corporation, Pittsburgh, PA, USA) and TI MSP430FR5994 microcontrollers (TEXAS INSTRUMENTS, Dallas, TX, USA) demonstrated that, at a distance of 1 m, 2.606 mJ could be harvested. This value exceeds the measured energy consumption of 1.416 mJ in active mode and 0.2175 mJ in low-power mode (LPM0), enabling sustainable battery-free operation. These results prove that the strategy is not only effective for detecting and localizing interference at the physical layer, but also represents a viable complement to spread spectrum defenses (DSSS, FHSS), providing an additional layer of resilience with low energy cost and deployability in critical infrastructures. Tran et al. (2020) in [37] examine how cooperative jamming and eavesdropping attacks impact communication reliability in energy-harvesting (EH) WSNs. In their model, cluster heads (CHs) supply energy to sensors and transmit data to the base station (BS) using non-orthogonal multiple access (NOMA), thereby improving spectral efficiency. To mitigate interference, the authors propose channel selection strategies and dynamic power allocation that ensure physical-layer security against information leakage. Results show that although increasing jammer power severely degrades link reliability, high levels of reliable communication (up to ∼95% in reference scenarios) can still be achieved by optimizing parameters such as EH time and CH power allocation. The study highlights that physical-layer defenses need not rely solely on spread spectrum techniques (DSSS, FHSS), but can be reinforced through energy- and channel-management policies, achieving a balance between security, energy efficiency, and resilience to jamming.

#### 2.1.8. Applications and Use-Case Scenarios of Jamming in WSNs

Beyond theoretical studies, jamming manifests differently depending on the application context. Industrial WSNs face interference from machinery, while WBANs must deal with proximity-based threats that directly affect human safety. Nanosensor networks and underwater WSNs present additional challenges due to unique propagation environments and stringent energy constraints. This theme highlights how application-driven requirements shape both the vulnerabilities and the solutions to jamming, underscoring the importance of tailoring strategies to real-world scenarios. By contextualizing research within these environments, studies provide practical insights for designing resilient, energy-aware WSNs.

Within the framework of jamming application scenarios in WSNs, the work of Sheela et al. (2023) in [51] offers a relevant example by proposing a CNN-based Quantum Leap detection method, capable of accurately identifying different types of interference (constant, random, deceptive, and reactive) using the WSN-DS dataset. The significance of this contribution lies in the fact that jamming attacks do not manifest uniformly but instead take on specific forms depending on the deployment context of the network: from industrial environments, where interference can disrupt critical processes, to health or security applications, where communication reliability directly impacts the protection of human life. In this regard, achieving 95.4% accuracy in attack classification addresses the need for adaptive and energy-efficient solutions that go beyond mere experimental validation and project themselves as strategies applicable to real-world environments. The work of Manju and Kumar (2012) in [52] represents an early and significant contribution by proposing a physical-layer identification method based on residual energy, the Receiver Signal Strength Indicator (RSSI), and the Packet Delivery Ratio (PDR). The validity of this approach is supported by simulation results in NS2, where 50 mobile nodes were deployed in a 750 × 750 m area, with a transmission range of 250 m and up to five attackers. The experiments compared the proposed method with the PDRSS technique (Packet Delivery Ratio and Signal Strength), an approach that detects jamming solely from these two basic metrics, showing that the proposed system consistently maintains higher throughput even as the number of attackers increases, thereby confirming its effectiveness in sustaining communication under adverse conditions. In this regard, the use of monitor nodes with greater residual energy to oversee and isolate potential attackers emerges as a practical strategy for real-world scenarios, where energy and topological constraints demand lightweight yet effective solutions. Complementary to earlier approaches focused on physical-layer identification or deep learning-based classification, the work of Liu et al. (2012) in [53] is particularly relevant as it introduces a two-phase detection scheme that balances two traditionally conflicting requirements: energy efficiency and timely detection. Unlike approaches relying exclusively on packet delivery ratio (PDR), which require frequent message exchanges and consequently high energy consumption, the authors propose a system in which the first phase relies on lightweight measurements of signal strength (RSSI) and overheard packet corruption rate (OPCR), triggering only when necessary a second, more costly phase based on PDR. Experimental results on TelosB motes demonstrate that the method achieves jamming detection in less than 0.5 s without false positives, while maintaining minimal resource usage. This finding is critical for sensitive applications such as border surveillance or critical infrastructure security, where reaction speed is as important as network longevity.

Hybrid approaches have gained increasing relevance in jamming and DoS detection within WSNs, as they combine lightweight monitoring with advanced ML to balance accuracy, efficiency, and adaptability. In this context, Premkumar and Sundararajan (2021) in [54] propose a defense framework against DoS attacks in WSNs using Deep Radial Basis Networks (DRBNs), combining a cluster-based hierarchical model with dynamic key management and bidirectional authentication to address threats such as jamming, flooding, black holes, and eavesdropping. Simulations in NS-2 with up to 200 nodes demonstrate a 94.6% detection rate, 98% accuracy, and a reduced FPR of 2.8%, along with lower energy consumption and latency compared to previous methods like MAS. These results highlight how hybrid deep learning approaches provide integral and scalable defenses, strengthening the resilience of WSNs in critical applications such as industrial automation, healthcare, and military surveillance. Within the framework of jamming application scenarios in WSNs, the work of López-Vilos et al. (2023) in [27] introduces an innovative approach by proposing the Fairness Cooperation with Power Allocation (FCPA) strategy, a clustering-based self-healing technique that integrates dynamic power allocation, node cooperation, and load balancing. The relevance of this contribution is evident when considering contexts such as agriculture, industry, or public safety, where jamming attacks can compromise both service continuity and the energy efficiency of resource-constrained devices. In simulations conducted with 64 static nodes under seven different attacker locations, FCPA achieved an average of 967,961 kb of information transmitted with 63% residual energy, outperforming other benchmark strategies by more than 50%. These findings demonstrate that schemes combining self-healing and clustering provide practical and adaptable solutions for real-world scenarios, such as WBANs, where node cooperation can safeguard patient safety against interference, or in industrial networks, where load balancing ensures operational continuity despite machinery-induced interference. The study by Zhang and Zhang (2019) in [55] is particularly illustrative, as it demonstrates that network vulnerabilities arise not only from external interference but also from internal threats such as the selective forwarding attack (SFA), which undermines reliable data delivery in critical contexts such as healthcare or defense. Their proposed scheme, E-watchdog, incorporates physical-layer parameters such as the SNR and leverages the collaboration of multiple detection agents, achieving a 25% reduction in false alarms and a 10% improvement in detection accuracy compared to the traditional watchdog mechanism, all with negligible energy overhead. Moreover, simulations with 100 deployed nodes and up to 10 malicious nodes show that packet delivery remains stable even under joint spoofing attacks, provided that node mobility does not exceed 21 m/s. Finally, in their study, Zhu and Jiang (2012) in [56] model the interaction between the attacker and the network as a noncooperative, non-zero-sum game, where the attacker adjusts its jamming probability (*q*) and the network regulates its channel access probability (γ). By employing a detection algorithm based on Wald’s Sequential Probability Ratio Test (SPRT) and the use of monitoring nodes, the authors evaluated configurations with a 20 m transmission range for sensor nodes and 200 m for the jammer, a node density of ρ=0.0025, and energy constraints of E/P=500 for sensor nodes and $E_{m}/P_{m}=1000$ for the attacker. The results reveal the existence of up to 16 Nash equilibria, among which those selected according to Pareto dominance maximize the payoffs of both parties, while those chosen under risk dominance provide greater stability under uncertainty.

To provide a consolidated view of prior research, Table 1 summarizes representative works on energy-aware jamming and anti-jamming strategies in wireless sensor networks, highlighting attack types, affected protocol layers, employed techniques, energy-related metrics, and reported limitations.

## 3. Materials and Methods

Building upon the thematic synthesis presented in the previous section, which examined how energy considerations intersect with jamming strategies, detection mechanisms, and mitigation techniques in WSNs, this section details the methodological framework adopted to systematize and validate the findings derived from the literature. While the related work highlighted the diversity of approaches and research directions, it also revealed the fragmentation of available studies in terms of energy modeling, evaluation metrics, and experimental reproducibility. To address this gap, an SLR was designed to identify, classify, and critically analyze the most relevant contributions dealing with the energy impact of jamming in WSNs. This approach ensures transparency and consistency in the process of selecting and interpreting prior studies, while also allowing a quantitative and qualitative assessment of existing research trends. The methodology described in this section follows an iterative and reproducible process comprising database selection, formulation of search strategies, and refinement of the resulting corpus through inclusion and exclusion criteria. Moreover, the search and filtering pipeline is formalized through a mathematical model of information search and refinement, which captures the optimization nature of literature selection. The following subsections describe, in sequence, the search methodology, the formal mathematical model supporting reproducibility, the protocol parameters that guide inclusion and exclusion decisions, and the database selection and screening process that led to the final set of studies included in this review.

### 3.1. Search Methodology

This section describes the methodology used to identify the literature and articles related to the focus of this research. The process is structured in accordance with standard guidelines for systematic reviews in engineering and computer science and comprises: (i) formulation of the research questions and aims, (ii) definition of inclusion and exclusion criteria, (iii) selection of databases, (iv) identification of search terms and query construction, and (v) multi-stage screening of titles, abstracts, and full texts. The objective of the SLR is to systematically review the literature on interference and jamming in wireless sensor networks, specifically from the perspective of the energy impact that such attacks cause on both the jammer and the network. The review aims to provide a comprehensive overview of the data, models, and algorithms used to analyze the energy impact of jamming in WSNs and to synthesize which strategies are reported as the most energy-efficient for both attacks and countermeasures.

The literature identified for the review was obtained through a stepwise process that began with database selection, identification of key search terms, formulation of search queries, and evaluation of the refined literature through title, abstract, and full-text screening. These steps are described in more detail below.

### 3.2. Mathematical Model for Information Search and Refinement

In order to ensure reproducibility and transparency, we model the information search and refinement procedure as an iterative optimization problem.

#### 3.2.1. Formal Search Space Definition

Let D denote the entire set of documents indexed in the selected bibliographic databases. A query q∈Q is defined as a Boolean or keyword-based search string constructed from conceptual terms $\left\{t_{1},t_{2},\ldots ,t_{k}\right\}$ belonging to three semantic categories:

C={“jammingattacks”,“energyapproach”,“wirelessnetworks”}.

The initial retrieved set of documents is expressed as:

$$R_{0}=\left\{d\in D|q\left(d\right)=1\right\},$$

where q(d) is an indicator function that evaluates to 1 if document *d* satisfies the query constraints, and 0 otherwise.

#### 3.2.2. Refinement Through Inclusion–Exclusion Criteria

To systematically refine the search space, we define two binary classifiers:

$$I\left(d\right)=\left\{\begin{array}{cc} 1, & \mathrm{if}\;d\;\mathrm{meets}\;\mathrm{all}\;\mathrm{inclusion}\;\mathrm{criteria},\\ 0, & \mathrm{otherwise}, \end{array}\right.\;E\left(d\right)=\left\{\begin{array}{cc} 1, & \mathrm{if}\;d\;\mathrm{meets}\;\mathrm{any}\;\mathrm{exclusion}\;\mathrm{criterion},\\ 0, & \mathrm{otherwise}. \end{array}\right.$$

The refined set of candidate documents after applying the criteria is:

$$R_{1}=\left\{d\in R_{0}|I\left(d\right)=1\wedge E\left(d\right)=0\right\}.$$

#### 3.2.3. Iterative Screening Process

The screening process is modeled as a sequence of filters:

$$R_{i+1}=f_{i}\left(R_{i}\right),\;i=1,2,\ldots ,n,$$

where $f_{i}\left(\cdot \right)$ corresponds to filtering at stage *i* (e.g., title screening, abstract screening, and full-text eligibility assessment).

At each stage, the reduction rate is defined as:

$$\delta_{i}=1-\frac{|R_{i+1}|}{|R_{i}|}.$$

The final set of included studies S is obtained as follows:

$$S=R_{n},$$

with |S|≪|D|, ensuring a focused and high-quality corpus of literature.

#### 3.2.4. Optimization Objective

The refinement process seeks to maximize the relevance of included studies while minimizing redundancy and noise. Formally, we aim to solve:

$$\max_{S\subseteq D}\;E\left[\mathrm{Relevance}\left(S\right)\right]-\lambda \cdot E\left[\mathrm{Noise}\left(S\right)\right],$$

where λ>0 is a trade-off parameter that penalizes the inclusion of low-quality or irrelevant studies.

This mathematical model formalizes the systematic review pipeline as an iterative search-and-refine mechanism, where query construction, inclusion/exclusion criteria, and progressive screening act as successive optimization filters. The resulting corpus ensures methodological rigor and validity in subsequent analyses.

While conventional systematic literature reviews typically describe search and screening stages procedurally (e.g., following PRISMA-style workflows), the formulation adopted in this work provides an explicit analytical abstraction of the refinement process. By modeling literature selection as an iterative optimization problem, the proposed approach makes the effects of inclusion criteria, screening stages, and reduction rates formally observable and reproducible.

This abstraction offers two practical advantages. First, it improves transparency by explicitly quantifying how successive filtering stages reduce the search space, rather than reporting screening outcomes descriptively. Second, it facilitates reproducibility and extensibility, as alternative queries, criteria, or screening functions can be incorporated within the same formal framework without altering the underlying methodology. This capability allows the refinement process itself to be examined, compared, and re-executed under alternative parameter settings, rather than treated as a fixed procedural outcome. As a result, the model supports objective comparison between different review configurations and reduces the reliance on reviewer-specific heuristics during literature refinement. To illustrate how the proposed formulation improves reproducibility, consider a common ambiguity in SLR workflows: studies that evaluate interference effects in IEEE 802.15.4 networks but do not explicitly label the phenomenon as “jamming.” In conventional SLR pipelines, the inclusion of such studies often depends on subjective interpretation of titles, abstracts, or keyword matches, leading to inconsistent decisions across reviewers.

**Figure 3:**
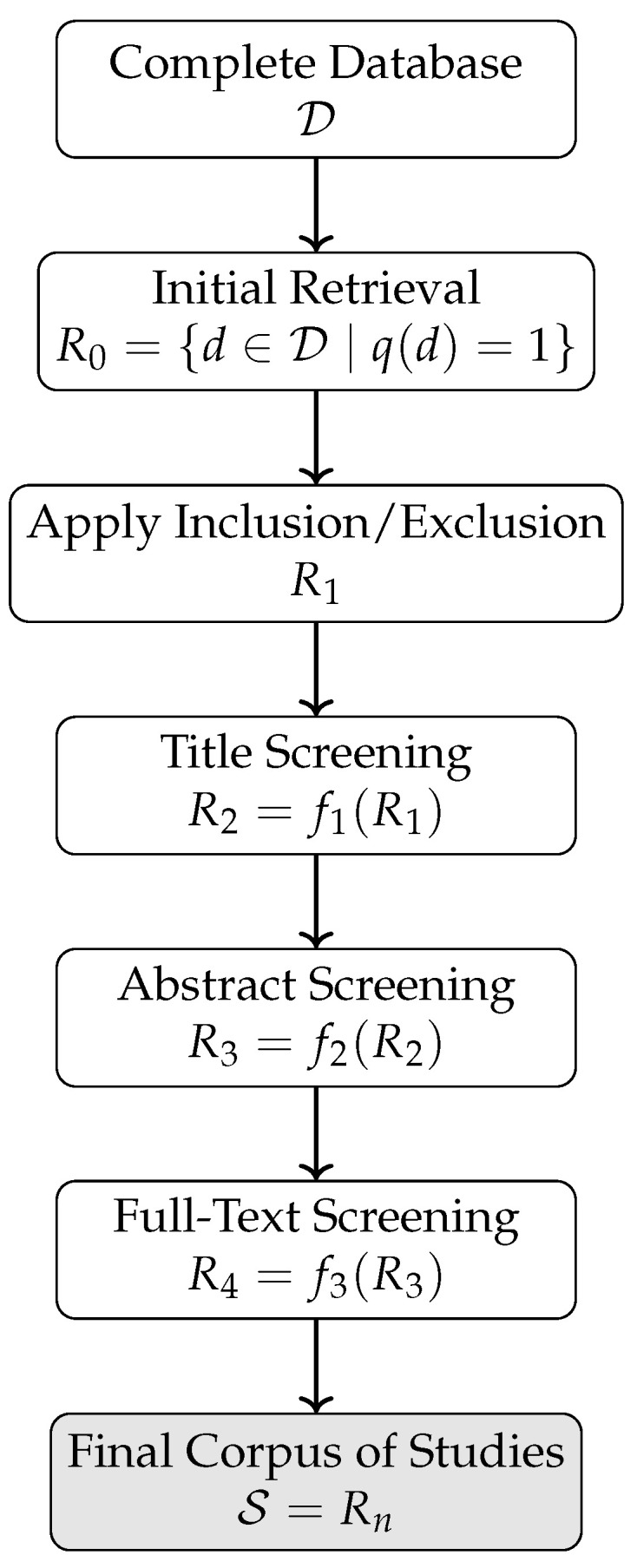
Flow diagram of the iterative search and refinement model.

Under the proposed search-and-refinement model, this ambiguity is resolved by construction. A study is retained if and only if it satisfies the formal constraints on (i) protocol compliance (IEEE 802.15.4), (ii) intentional or sustained interference affecting communication availability, and (iii) explicit evaluation of energy-related performance metrics. Terminological differences, therefore, do not affect inclusion, as selection is driven by constraint satisfaction rather than semantic interpretation. This mechanism ensures that identical inputs yield identical inclusion outcomes, thereby improving transparency and reproducibility in borderline cases. This example illustrates how the proposed formulation addresses a recurrent source of subjectivity in systematic reviews of wireless security, particularly in domains where terminology and experimental intent are not consistently aligned. The workflow of the search and refinement model is illustrated in Figure 3.

#### 3.2.5. Protocol Implemented

This includes specifying the research question, the objective of the research project, the objective of the systematic literature review, and the inclusion and exclusion criteria. Table 2 defines each of the parameters necessary for the definition of the implemented protocol.

#### 3.2.6. Database Selection

The systematic literature review was conducted rigorously between March and July 2025, utilizing the Scopus database to ensure the reliability and pertinence of the findings. Scopus was selected as the sole indexing source because it functions as an independent and multidisciplinary scientific indexer that consolidates peer-reviewed publications from major publishers, including IEEE, ACM, Elsevier, MDPI, Springer, and Wiley. Unlike publisher-controlled repositories, whose coverage is restricted to their own editorial output and which do not operate as formal scientific indexers, Scopus provides neutral and comprehensive access to a broad spectrum of scientific literature. Its inclusion standards involve quality assessment, metadata normalization, and cross-publisher deduplication, which enhances the consistency and rigor of the retrieved material.

To ensure that the use of a single indexer did not introduce selection bias, preliminary cross-checks were performed in IEEE Xplore and ACM Digital Library. All publications retrieved in these repositories that matched the defined search terms were already indexed in Scopus, confirming complete coverage for the scope of this review. Because Scopus aggregates metadata from these major publishers, and no additional relevant studies were found outside its index, using Scopus as the sole source did not exclude pertinent literature.

#### 3.2.7. Identification of Search Terms

Three key conceptual terms were identified for the systematic literature review: jamming attacks, energy approach, and wireless networks. All supplementary search terms leveraged in constructing the search query correspond to one of these core conceptual categories.

#### 3.2.8. Search Query

The search phrase was structured as follows “(“jamming attacks*” OR “interference attacks*”) AND (“energy approach*” OR “energy efficient*” OR “energy harvesting*” OR “energy impact*” OR “energy expenditure*” OR “power consumption*”) AND (“node” OR “jammer” OR “wireless networks*” OR “WSN”)”. This search was refined explicitly by focusing on the title, abstract, and keywords of relevant publications, resulting in an initial identification of 482 articles.

### 3.3. Study Selection Results

The initial search identified a total of 482 distinct articles ($R_{0}$). The selection process then followed the staged refinement model described in Section 3.2 First, the inclusion and exclusion criteria defined in Table 2 were applied to the raw set, which led to the exclusion of 76 articles that did not satisfy the language, time window, or peer-review requirements. This step resulted in $R_{1}=406$ articles.

In the next stage, titles were screened to discard studies that were clearly unrelated to jamming or energy-aware WSNs, removing 125 articles and leaving $R_{2}=281$ records. Abstract screening was then performed to assess conceptual and methodological relevance in more detail, which led to the exclusion of 130 additional articles and yielded $R_{3}=151$ candidates. Finally, full-text eligibility assessment was conducted on these 151 articles, excluding 89 that did not meet the energy-centric focus or lacked sufficient methodological detail regarding energy modeling, evaluation metrics, or jamming strategies. The remaining 62 articles constituted the final corpus of included studies, i.e., $S=R_{4}$.

**Figure 4:**
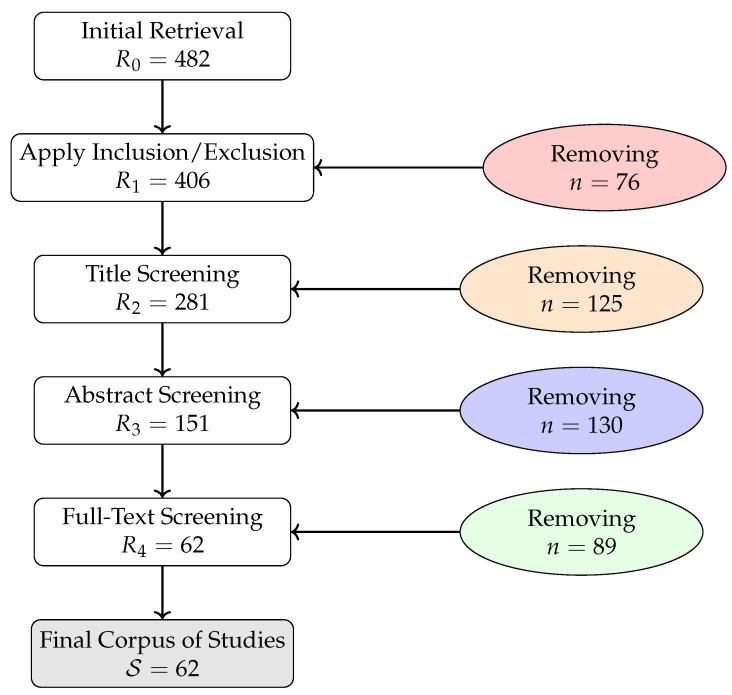
Flow diagram of the iterative search and refinement model, including removed records at each stage.

A detailed flow diagram outlining the selection process, including the number of excluded records at each stage, is provided in Figure 4.

#### 3.3.1. Data Extraction and Thematic Coding

For each included publication, a structured data extraction form was completed to collect qualitative and quantitative attributes relevant to energy-aware jamming analysis. The extracted fields included: (i) type of jamming attack (constant, random, reactive, protocol-aware, selective), (ii) network layer affected (PHY, MAC, routing, cross-layer), (iii) jamming or anti-jamming technique (e.g., power control, channel hopping, duty-cycling adaptation, ML-based detection), (iv) reported energy metrics (e.g., current consumption, joules per transmission, duty cycle, battery lifetime, energy harvesting model), (v) evaluation method (simulation, analytical model, testbed), (vi) hardware or simulation platform (e.g., TelosB, CC2420, CC1352, NS-2/3, OMNeT++, MATLAB), with software version information recorded when explicitly reported in the original studies, and (vii) performance outcomes and reported limitations.

These variables enabled a consistent comparison of studies and supported the construction of the synthesis tables presented in Section 4. The qualitative fields (attack type, affected layer, mitigation approach) were used to assign each paper to one or more of the eight thematic domains defined in Section 2. The coding was performed by the primary reviewer and cross-checked by a second author; disagreements were resolved through discussion. This thematic classification enabled cross-layer synthesis and the identification of methodological and energy-related gaps in Section 4 and Section 5.

Unlike prior surveys on jamming and anti-jamming in IEEE 802.15.4-based wireless sensor networks, which primarily organize attacks and defenses according to protocol layer, attack modality, or detection technique, the classification adopted in this review is explicitly structured around energy as the central analytical dimension. Rather than treating energy consumption as a secondary performance indicator, the adopted taxonomy organizes jamming strategies and countermeasures according to their energy behavior, energy asymmetry, and long-term sustainability implications.

A second distinguishing aspect of this classification lies in its implicit joint consideration of attacker and defender energy dynamics. While most existing surveys analyze jamming effectiveness and defensive performance in isolation, the adopted taxonomy emphasizes how attacker energy expenditure, defender overhead, and protocol behavior interact over time, enabling a more balanced interpretation of resilience under sustained interference.

Finally, the taxonomy explicitly incorporates cross-layer effects and recurring experimental assumptions, such as MAC behavior, duty cycling, traffic patterns, and evaluation platforms, as first-order elements of classification. This energy-centric and assumption-aware perspective provides a structured basis for interpreting the heterogeneous results synthesized in the following section.

#### 3.3.2. Study Quality Considerations

Since all included publications were peer-reviewed conference or journal articles indexed in Scopus, no additional formal quality appraisal scheme (such as CASP or JBI tools) was applied. Instead, methodological transparency, completeness of energy reporting, and clarity of experimental setup were considered implicit indicators of study quality. Studies lacking sufficient methodological detail or not reporting energy-related metrics in a traceable way were excluded during the screening phases described above.

#### 3.3.3. Growth of Scholarly Literature and Its Geographical Dissemination Across Countries

**Figure 5:**
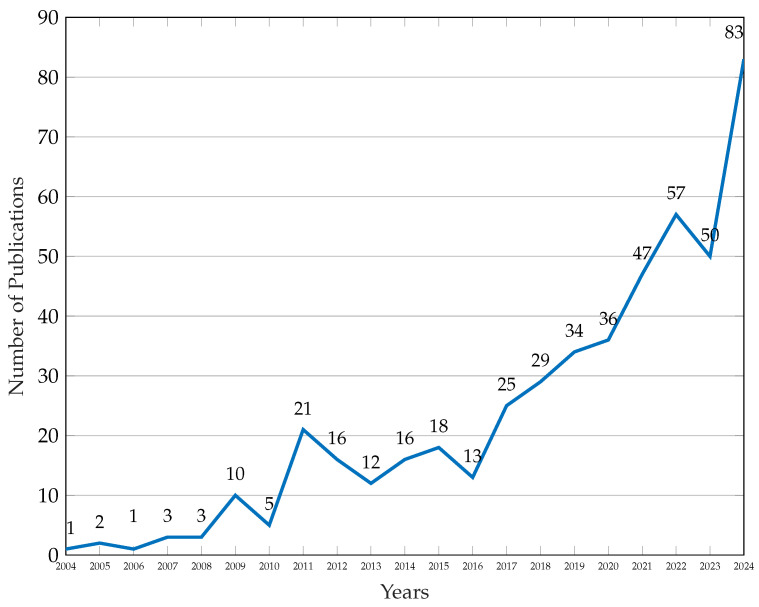
Publications trend from 2004 to 2024.

The analysis reveals a notable trend in publication volume, beginning with a modest output of 1 or 2 papers annually from 2004. However, there was a significant surge in publications starting around 2011, as illustrated in Figure 5. This upward trajectory suggests a growing interest in the field post-2011.

**Figure 6:**
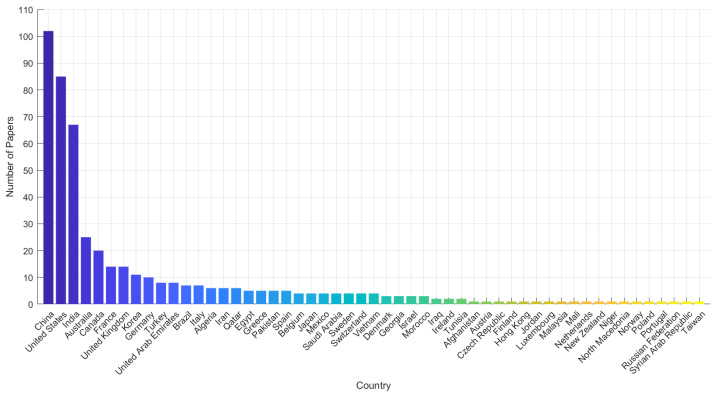
Academic articles by country according to the initial search.

Figure 6 also shows the countries of origin of the publications. China leads with 102/482 publications of the total contributions, followed by the United States and India with 85/482 and 65/482, respectively. In addition, Australia and Canada are fourth and fifth in the ranking with 25/482 publications and 20/482 contributions, respectively.

#### 3.3.4. Number and Distribution of Relevant Articles

**Figure 7:**
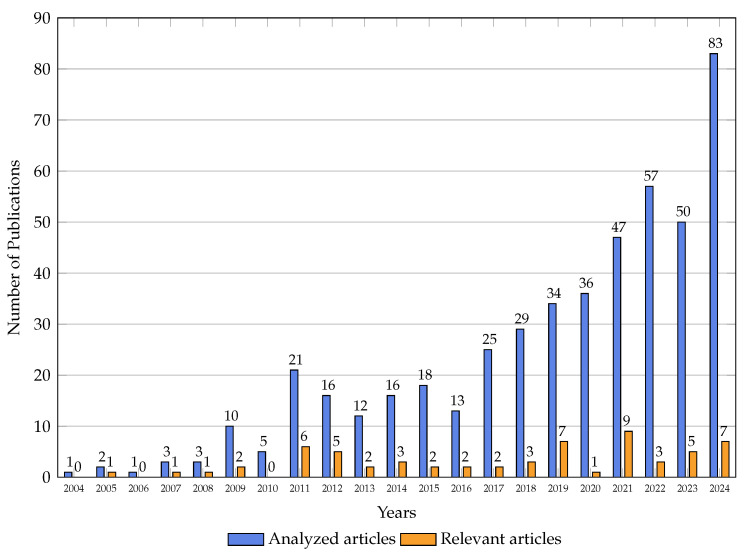
Comparison of the total analyzed publications versus the count of pertinent studies focused on jamming attacks in WSNs employing an energy-centric approach from 2004 to 2024.

The annual distribution of both the total number of collected articles and those deemed relevant for this review is illustrated in Figure 7. While the data indicates a rising trend in the quantity of published articles over time, the increase in relevant articles does not correspond proportionally. This discrepancy implies that, despite the growing volume of articles, a significant portion fails to align with the relevance criteria established for this review.

#### 3.3.5. Analyzing the Influence and Interrelationship Among the Most Significant Articles

**Figure 8:**
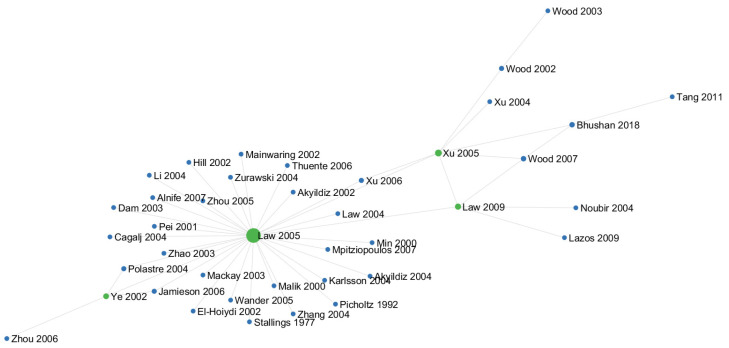
Citation network illustrating structural relationships among early and transitional contributions in the jamming and anti-jamming literature for wireless sensor networks.

The citation network in Figure 8 highlights seminal contributions that structurally anchor the contemporary literature on jamming and anti-jamming in wireless sensor networks, with the work by Law et al. [57] clearly emerging as a dominant intellectual hub. Its central position reflects how early cross-layer analyses of denial-of-service and jamming attacks consolidated a shared research foundation spanning PHY-, MAC-, and network-layer perspectives. The dense set of connections surrounding this node indicates a consolidating role within an early citation cluster that includes foundational contributions on wireless sensor network architecture, interference-prone communication, and MAC-layer robustness [1,58,59], rather than a unidirectional line of influence. In this sense, prior studies addressing system-level design considerations, empirical packet delivery limitations, and availability-oriented protocol behavior converge in this early body of work, which subsequently emerges as a key reference point for later research on adversarial interference and jamming. A second tier of influential nodes, including Xu et al. [5] and the extended journal version of this work [21], marks a gradual transition toward more adaptive and selective jamming models, where energy efficiency and attacker–defender asymmetry begin to play a more explicit role in shaping threat models and mitigation strategies. This transition signals a shift from coarse-grained, availability-centered analyses toward energy-aware interpretations of jamming impact, enabling later studies to assess disruption not only in terms of packet loss, but also in accelerated energy depletion and reduced network lifetime.

## 4. Analytical Framework for Results Interpretation

This section establishes the interpretative lens through which the results of the systematic literature review are synthesized. The findings reported in the following section are analyzed in light of the system model and scope defined for IEEE 802.15.4-based wireless sensor networks, the CIA-oriented threat perspective that frames jamming as an availability- and energy-depletion attack, and the energy-centric classification adopted in this review.

By grounding the analysis in these complementary elements, the results are interpreted not merely as isolated observations, but as structured patterns that reflect the interaction between attacker strategies, defensive mechanisms, and underlying protocol assumptions. This framing supports a consistent and comparable interpretation of energy-related behaviors across the reviewed studies and provides the conceptual basis for identifying cross-cutting trends, trade-offs, and open research challenges.

## 5. Results

The results of the review reveal how energy considerations shape both offensive and defensive dynamics in wireless sensor networks. To contextualize these findings, the section begins with a quantitative summary of the 62 selected studies, highlighting the evolution of publication trends and the thematic focus of the corpus. Subsequent subsections detail the observed patterns in attacker energy consumption, defender overhead, and cross-layer or energy-harvesting solutions, as well as the key research challenges identified across the literature.

### 5.1. Quantitative Overview of the Selected Literature

The final dataset comprises 62 peer-reviewed studies published between 2004 and 2024. A marked increase in research activity is observed in recent years: 39 studies (≈63%) were published from 2016 onward, and 25 studies (≈40%) correspond to the 2020–2024 period, reflecting a growing interest in energy-aware jamming research as WSNs adopt ultra-low-power and duty-cycled architectures.

With respect to document type, the dataset includes 33 conference papers (≈53%), 28 journal articles (≈45%), and 1 review article (≈2%), indicating that the topic remains strongly driven by emerging methods and conference-level experimentation. A textual analysis of abstracts reveals that jamming is explicitly mentioned in 51 studies (≈82%), while *energy* appears in 61 studies (≈98%), confirming that the intersection between interference and energy consumption constitutes the central thematic axis of this body of work. This quantitative characterization reinforces the relevance of analyzing jamming not only as a security problem but also as an energy-governed phenomenon affecting the operational sustainability of WSNs.

### 5.2. Energy-Related Performance Metrics

To operationalize the proposed energy-centric taxonomy, Table 3 and Table 4 explicitly map each reviewed study to the core taxonomy dimensions introduced in Section 2, thereby enabling systematic comparison across studies. In particular, the columns Attack Type and Affected Layer(s) instantiate the jamming-modality and protocol-layer dimensions, while Technique/Protocol captures the concrete realization of attacks or countermeasures. The Energy Metric column operationalizes the energy-centric dimension by making explicit how energy consumption and depletion are quantified across studies. Finally, the Key Findings and Limitations columns contextualize these dimensions by reporting network-level effects, energy asymmetries, and methodological constraints under the experimental assumptions summarized in Table 4.

To facilitate a consistent interpretation of the energy-related findings reported across the reviewed studies, it is necessary to clarify the performance metrics most commonly used to characterize jamming and anti-jamming behavior in IEEE 802.15.4-based wireless sensor networks. While the literature exhibits considerable heterogeneity in how energy consumption and availability are quantified, a recurring subset of metrics emerges as both practical and informative for capturing the energy impact of interference.

In particular, the surveyed works predominantly rely on a small number of complementary indicators to assess energy efficiency and network robustness under jamming conditions. These include energy per successfully delivered packet or bit, radio-on time or duty cycle as proxies for sustained energy drain, network or node lifetime with explicitly stated failure criteria, and packet delivery ratio as a primary availability metric. When available, the reporting of attacker energy consumption further enables the analysis of energy asymmetry between adversarial and defensive strategies, providing additional insight into long-term network sustainability.

Table 3 summarizes these energy-related performance metrics and their typical roles in jamming-oriented evaluations, establishing a common reference framework for the subsequent analysis of attacker behaviors, defensive overhead, and cross-layer mitigation strategies.

To further strengthen the joint attacker–defender perspective emphasized in this review, we introduce a minimal attacker–defender energy asymmetry indicator as an interpretative construct. Conceptually, this indicator relates the energy expended by the attacker to the resulting availability degradation observed at the defender side, where availability degradation is captured through commonly reported metrics such as packet delivery ratio reduction, increased retransmission rates, prolonged radio-on time, or accelerated node or network lifetime depletion.

Rather than serving as a strict numerical benchmark, this asymmetry indicator provides a consistent lens for interpreting how efficiently an attacker converts its own energy expenditure into availability loss and energy drain within the network. Importantly, existing studies can be mapped to this indicator even when only partial metrics are reported. For instance, works that report attacker transmission energy together with defender-side PDR degradation or radio-on time implicitly characterize this asymmetry, while studies reporting only defender lifetime reduction under sustained jamming capture the defender-side impact component of the same relationship.

By grounding this asymmetry indicator in the energy-related metrics summarized in Table 3, the proposed formulation enables conceptual cross-study comparison without imposing additional measurement requirements or reprocessing original experimental data. This reinforces the operational role of the energy-centric taxonomy and supports a unified interpretation of attacker efficiency and defender energy cost across heterogeneous evaluation settings. Furthermore, this structure enables new or future studies to be systematically positioned within the proposed taxonomy by mapping their experimental assumptions and reported metrics directly onto the same dimensions, thereby supporting reproducible and taxonomy-driven comparison beyond the specific corpus analyzed in this review.

### 5.3. Attack-Side Energy Patterns

Building on the attacker–defender energy asymmetry identified in Section 5.2, attacker-side energy patterns reveal a fundamental trade-off between disruption persistence and energy efficiency. Several studies explicitly examine how different jamming modalities influence the attacker’s energy expenditure. A consistent finding is that constant jamming is the most energy-intensive strategy, as its continuous transmission behavior rapidly depletes attacker energy reserves and exerts sustained pressure on nearby sensor nodes. By contrast, reactive and slot-based jammers activate only upon detecting legitimate communication or predetermined temporal conditions, demonstrating substantially improved disruption-to-energy ratios. Some works also present probabilistic or intermittent jamming techniques, which reduce active transmission time while preserving disruptive capacity. This trend reflects a broader evolution in adversarial modeling, in which attackers increasingly adopt energy-adaptive interference patterns aligned with the duty-cycling and low-power operation of modern WSNs. Rather than relying on brute-force interference, contemporary jamming strategies in the literature emphasize energy efficiency, selective activation, and protocol-aligned timing.

Across the reviewed studies, attacker-side energy patterns reveal a fundamental trade-off between disruption persistence and energy efficiency. While constant jamming maximizes immediate availability degradation, its continuous transmission behavior results in rapid attacker energy depletion, limiting long-term effectiveness. In contrast, reactive, selective, and slot-based jammers achieve higher disruption-to-energy ratios by aligning interference activity with legitimate transmissions or protocol timing, thereby conserving attacker energy while maintaining comparable impact on network availability. However, this efficiency comes at the cost of increased complexity, including sensing accuracy requirements, protocol awareness, and tighter synchronization, which may constrain feasibility in highly dynamic or heterogeneous deployments.

### 5.4. Defense-Side Energy Overhead

From the defender’s perspective, and in line with the attacker–defender energy asymmetry discussed in Section 5.2, the reviewed studies reveal wide variation in the energy cost associated with anti-jamming techniques:

1. MAC-layer protocols demonstrate distinct resilience profiles. Duty-cycled protocols such as T-MAC may incur additional overhead depending on interference intensity, while fixed-schedule protocols like LMAC can reduce idle listening under moderate jamming.
2. Lightweight detection schemes, such as LADE, exhibit extremely low overhead (∼0.3%), representing one of the most energy-efficient mitigation techniques reported in the literature.
3. Feature-based detectors relying on RSSI variance, waveform entropy, or wavelet-energy descriptors may impose higher sampling and processing requirements, depending on feature dimensionality and observation windows.
4. At the routing layer, clustering-based approaches and fairness-aware power control mechanisms, such as FCPA, report high residual-energy retention (up to 63%), enhancing network lifetime under sustained jamming.

Collectively, these findings show that modern defenses increasingly prioritize energy proportionality, where mitigation intensity adapts to network energy availability rather than applying uniform or brute-force countermeasures.

Building on these observations, defender-side countermeasures exhibit a complementary set of energy trade-offs, primarily balancing mitigation effectiveness against proportional energy overhead. Lightweight detection schemes and adaptive MAC-layer mechanisms generally preserve energy efficiency by minimizing additional sensing, processing, or retransmission costs, but may offer limited protection under highly adaptive or persistent jamming. Conversely, more sophisticated defenses, including feature-based detection, cross-layer coordination, and learning-driven approaches, can significantly improve detection accuracy and availability restoration, at the cost of increased computational and communication overhead. The reviewed literature indicates a growing shift toward energy-proportional defenses, in which mitigation intensity adapts to perceived threat level or residual energy, rather than relying on static or always-on protection. This trend underscores that no single defense dominates across scenarios; instead, energy-aware resilience emerges from carefully tuning the trade-off between robustness, complexity, and long-term network sustainability.

### 5.5. Cross-Layer Mechanisms and Energy-Harvesting Approaches

As previously discussed in Section 5.4, cross-layer interactions between protocol layers play a central role in shaping energy consumption and resilience under jamming. A smaller subset of the literature investigates cross-layer solutions and energy-harvesting mechanisms as strategies for increasing resilience under jamming:

1. Cross-layer designs integrating PHY-layer detection with MAC-layer coordination help reduce idle listening, redundant sampling, and unnecessary retransmissions, yielding measurable energy savings.
2. Energy-harvesting-enabled systems demonstrate the ability to partially offset jamming-induced overhead, particularly in solar-based deployments where environmental availability allows partial recovery of consumed energy.
3. Machine learning-based detection, including lightweight CNN architectures, achieves high accuracy (often > 90%) while maintaining compatibility with embedded hardware constraints.

These studies highlight the importance of energy-autonomous and data-adaptive defensive architectures, especially as WSNs evolve toward long-lived, self-sustaining deployments in adversarial RF conditions.

Beyond the diversity of reported energy metrics, the reviewed studies also rely on a set of recurring experimental assumptions that strongly influence how jamming impact and energy consumption are evaluated. These assumptions, which range from channel and traffic models to MAC configurations and jammer behavior, directly shape both the reported energy outcomes and the comparability of results across studies.

To make these underlying conditions explicit, Table 4 summarizes the most common experimental assumptions observed in IEEE 802.15.4-based energy-aware jamming and anti-jamming evaluations, together with their implications for energy interpretation. By consolidating these assumptions, the table provides additional context for the energy patterns discussed in the preceding subsections and clarifies the sources of variability that motivate the research gaps identified in the next subsection.

### 5.6. Identified Gaps and Open Research Challenges

The experimental assumptions summarized in Table 4 provide important context for interpreting the limitations observed across the reviewed studies. These limitations are particularly pronounced when considering the gap between simulation-based assumptions and real-world hardware behavior.

Beyond the prevalence of simulation-based evaluations, the reviewed literature recurrently omits several testbed-level elements that materially affect energy-related conclusions and reported asymmetries under jamming. First, fine-grained hardware power-state behavior is rarely captured, including transient states such as radio wake-up, carrier sensing, and state transitions between sleep, idle, receive, and transmit modes. These transient phases can contribute significantly to energy consumption in IEEE 802.15.4 devices, particularly under reactive or intermittent jamming.

Second, many studies assume idealized duty-cycling schedules with perfectly periodic timing and negligible clock drift. In practical deployments, timing jitter and asynchronous wake-up behavior influence idle listening duration, retransmission probability, and susceptibility to timing-aligned jamming, directly impacting measured energy efficiency. Third, jammer sensing and synchronization behavior is often simplified, with reactive or selective jammers modeled as having instantaneous or error-free detection. In real hardware, sensing latency and synchronization overhead affect both attacker energy expenditure and disruption effectiveness, potentially altering observed attacker–defender energy asymmetries.

While these assumptions enable tractable analysis and facilitate early-stage evaluation, they also introduce methodological constraints that directly shape the scope, comparability, and reproducibility of reported energy-aware jamming results. The following gaps emerge consistently from the interaction between these assumptions and the evaluated jamming and anti-jamming mechanisms.

Despite the progress documented across the selected studies, several methodological gaps persist:

- Heterogeneous energy metrics: As reflected in the diverse energy models and reporting practices summarized in Table 4, studies report energy using non-standardized units (e.g., Joules, current draw, battery percentage, or estimated lifetime), which limits cross-paper comparability.
- Limited joint evaluation of attacker and defender energy: Consistent with the typical evaluation platforms and jammer configurations outlined in Table 4, only a minority of studies jointly analyze attacker and defender energy consumption, despite the centrality of this asymmetry for long-term resilience assessment.
- Prevalence of simulation-only evaluations: As indicated by the evaluation platforms summarized in Table 4, the majority of studies rely on simulation-based tools (e.g., NS-2, NS-3, OMNeT++), with relatively few testbed-based validations, limiting the realism of reported energy metrics.
- Incomplete characterization of jamming intensity: In line with the simplified jammer behavior and power assumptions reported in Table 4, key parameters such as jammer duty cycle, transmission power, and spectral occupancy are often underreported, reducing reproducibility and cross-study comparison.
- Underexplored energy-harvesting dynamics: As suggested by the simplified energy models summarized in Table 4, relatively few studies capture the temporal variability and environmental dependence of energy-harvesting processes, particularly under sustained or adaptive jamming conditions.

These gaps underscore the need for standardized benchmarking, multi-dimensional evaluation frameworks, and joint attacker–defender energy modeling to advance the field toward more reproducible and analytically robust methodologies. Collectively, the omission of these testbed elements can lead to systematic underestimation or mischaracterization of energy costs under jamming, particularly for reactive and protocol-aware attacks. As a result, conclusions drawn solely from simulation-based evaluations may overstate defensive efficiency or attacker effectiveness when translated to real deployments, underscoring the need for hardware-aware validation in future energy-centric jamming studies.

### 5.7. Methodological Trends in Energy-Aware Jamming Research

The temporal distribution of publications indicates a clear methodological transition after 2015. As WSNs increasingly adopt ultra-low-power designs, research on jamming has expanded from traditional interference modeling to energy-centric analyses, emphasizing cross-layer coordination, optimization-based formulations, and machine learning-driven detection. The methodological rigor of this review aligns with that evolution. By structuring the literature selection as a relevance-oriented filtering process, the review minimizes redundancy and maximizes informational value, mirroring the principles of energy-efficient system design. The resulting synthesis provides a coherent foundation for future research integrating energy theory, adversarial modeling, and computational intelligence within unified analytical frameworks.

## 6. Discussion

The following discussion interprets the results presented in the previous section by examining how the observed patterns relate to the operational behavior of wireless sensor networks under adversarial interference. Rather than reiterating the findings, this section explores their broader implications, identifies the interactions that shape energy-aware resilience, and highlights how emerging techniques influence the design and sustainability of defensive strategies. The analysis is organized into thematic components that collectively deepen the understanding of how energy dynamics influence both attackers and defenders.

### 6.1. Energy as a Bidirectional Constraint in Jamming Dynamics

The evidence reviewed in this study shows that energy governs both offensive and defensive behaviors in WSNs, but not in a symmetrical manner. While attackers can adopt high-duty or continuous-interference strategies without strict operational penalties, battery-powered sensor nodes must absorb the energy cost of detection, retransmissions, idle listening, and route reconfigurations. This imbalance clarifies why energy-efficient jamming techniques, such as reactive or time-aligned interference, pose challenges that extend beyond traditional security considerations. The findings indicate that resilience cannot be fully understood merely by characterizing the type of jamming; instead, it must incorporate how each interference pattern reshapes the energy trajectory of the network.

This interpretation provides a complementary perspective to the energy-aware taxonomy proposed earlier in the paper. Instead of categorizing techniques as isolated entities, the discussion highlights how attacker and defender energy constraints interact, revealing opportunities for targeted mitigations that are grounded in the real operational asymmetry of WSN platforms.

### 6.2. Cross-Layer Trade-Offs and Redistribution of Energy Costs

The results show that resilience mechanisms distribute energy costs differently depending on where they operate in the protocol stack. PHY-layer robustness often reduces immediate vulnerability but may trigger additional MAC-layer activity or complex synchronization events. MAC-layer improvements, such as duty-cycling and adaptive contention reduction, lower idle listening but may cause longer-term routing fluctuations. Network-layer approaches help maintain connectivity under interference but regularly introduce cluster maintenance or additional control packets.

This multi-layer redistribution of costs suggests that the resilience of a WSN under jamming is heavily shaped by interlayer feedback loops. For example, a defense that reduces retransmissions at the MAC layer may inadvertently increase control traffic at the network layer, or vice versa. The findings therefore reinforce the value of integrated designs that explicitly account for these interactions rather than optimizing each layer independently. This interpretation underscores how cross-layer coupling shapes energy consumption and operational sustainability, offering a broader systems-level perspective on resilience under jamming.

### 6.3. Critical Trade-Offs in Energy-Aware Jamming Mitigation

Although the reviewed literature reports a wide range of jamming mitigation strategies, the results reveal that no single approach provides uniform benefits across all deployment scenarios. Instead, energy-aware resilience emerges as a balance between protection effectiveness, energy overhead, and operational predictability. Techniques that significantly improve resistance to interference often do so at the cost of increased energy consumption, protocol complexity, or reduced network stability.

At the PHY and MAC layers, aggressive countermeasures such as frequent channel hopping, continuous monitoring, or fine-grained synchronization can suppress jamming effects but tend to increase radio-on time and processing load. Conversely, lightweight mechanisms, including probabilistic detection or duty-cycling adaptation, reduce energy expenditure but may allow intermittent service degradation under reactive or protocol-aware jamming. This trade-off indicates that maximizing availability does not necessarily maximize network lifetime.

Routing and network-layer defenses further illustrate this tension. While clustering and path redundancy can preserve connectivity under sustained interference, they introduce control overhead and reconfiguration costs that may outweigh their benefits in low-traffic or sparsely deployed networks. Similarly, cross-layer approaches improve coordination between detection and mitigation, but their effectiveness depends on accurate parameter tuning and predictable traffic patterns, which are rarely guaranteed in real deployments.

Overall, the evidence suggests that energy-aware jamming mitigation should not be evaluated solely by attack suppression, but rather through a multi-dimensional lens that considers energy proportionality, adaptability, and long-term sustainability. Explicitly acknowledging these trade-offs enables a more realistic assessment of resilience strategies and provides actionable guidance for selecting defenses that align with application-specific energy constraints.

### 6.4. Emerging Roles of Energy Harvesting and Machine Learning

Energy harvesting and machine learning have emerged as promising complementary approaches for improving resilience against jamming in wireless sensor networks. Energy harvesting techniques aim to mitigate the impact of energy depletion by enabling nodes to replenish their power from environmental sources, such as solar, vibrational, or radio-frequency energy. In jamming scenarios, these approaches can extend network lifetime and partially offset the increased energy expenditure induced by sustained interference, although their effectiveness remains strongly dependent on environmental conditions and harvesting stability.

In parallel, machine learning-based mechanisms have been increasingly explored for jamming detection, classification, and adaptive response. By learning traffic patterns, signal characteristics, or interference behaviors, these techniques can improve detection accuracy and enable more adaptive mitigation strategies. However, their deployment introduces additional computational and communication overhead, which must be carefully balanced against the energy constraints inherent to wireless sensor networks.

In addition to energy harvesting and machine learning-based approaches, it is important to acknowledge that a broader class of physical-layer security mechanisms has been widely studied in wireless communications. These include physical-layer authentication techniques as well as legacy physical-layer security methods such as beamforming and artificial noise injection. Such approaches are primarily designed to enhance confidentiality and integrity by exploiting channel characteristics and spatial selectivity, rather than to mitigate availability-oriented attacks.

In the context of jamming, physical-layer authentication and legacy PLS techniques are generally complementary, as they do not directly address energy depletion or sustained service disruption caused by interference. For this reason, while these mechanisms are recognized as part of the broader wireless security landscape, the present review focuses on energy-centric and availability-driven mitigation strategies that are more directly aligned with jamming resilience in wireless sensor networks.

### 6.5. Methodological Blind Spots and Their Impact on Interpretation

The systematic analysis confirmed several limitations that affect how results in the field should be interpreted. The heterogeneity in energy metrics complicates the comparison of reported gains or efficiencies across studies. Likewise, the predominance of simulation-based evaluations, combined with scarce hardware-level measurements, limits the generalizability of several proposed techniques. A particularly significant observation is that many studies characterize jamming intensity only partially, omitting parameters that are essential for understanding energy behavior, such as jammer duty cycle or spectral occupancy. Without these details, interpreting the magnitude of reported improvements becomes challenging, especially when comparing across different environments or hardware platforms. By explicitly connecting methodological inconsistencies with interpretive risks, this analysis adds a layer of critical assessment that strengthens the credibility of the review.

### 6.6. Implications for Representative IEEE 802.15.4 Deployment Scenarios

The energy-centric patterns and trade-offs identified in this review can be directly interpreted in the context of common IEEE 802.15.4 deployment scenarios. In industrial monitoring and control applications, where star or tree-based topologies and periodic traffic are prevalent, constant or high-duty jamming can rapidly degrade availability by forcing repeated retransmissions and prolonged radio activity. In such settings, lightweight detection mechanisms combined with duty-cycling-aware MAC adaptations offer a favorable balance between resilience and energy consumption, particularly when strict latency constraints are present.

In smart building and home automation deployments, which typically operate under low traffic intensity and aggressive duty cycling, reactive or protocol-aware jamming poses a greater challenge than continuous interference. Here, defenses that rely on adaptive wake-up scheduling or probabilistic detection may tolerate brief service degradation while preserving long-term node lifetime. Cross-layer coordination is especially relevant in these scenarios, as small increases in control overhead can disproportionately affect battery-powered devices.

Healthcare and wearable sensing systems emphasize availability and predictability while operating under severe energy constraints. In these deployments, excessive mitigation overhead may be as detrimental as the jamming itself. The reviewed evidence suggests that conservative, energy-proportional defenses and early detection strategies are better suited to such applications than aggressive channel or routing reconfiguration.

Overall, this mapping highlights that the effectiveness of energy-aware jamming mitigation strategies is inherently scenario-dependent. Selecting appropriate defenses requires aligning mitigation intensity, protocol behavior, and energy availability with the operational characteristics of the target IEEE 802.15.4 deployment, rather than applying uniform countermeasures across heterogeneous application domains.

### 6.7. Implications for Future Research Directions

The insights derived from this analysis point toward several opportunities for advancing energy-aware jamming research. The results suggest that next-generation WSNs will require tuning of resilience mechanisms not only to attack characteristics but also to energy availability profiles, environmental conditions, and protocol-layer interactions. Integrating lightweight ML models with adaptive MAC and energy-harvesting-aware scheduling could yield architectures capable of modulating defensive actions in real time.

Furthermore, cross-layer optimization appears to be a promising direction, especially for resource-constrained deployments where the redistribution of energy burdens must be predictable and controllable. The findings also imply a need for experimental setups that capture the combined influence of hardware characteristics, harvesting variability, and real interference, elements that remain underrepresented in simulation-based studies. This discussion underscores how the collected evidence shapes practical design implications and indicates where the research community should focus to address remaining conceptual and empirical gaps.

## 7. Conclusions

This review shows that energy awareness plays a central role in shaping how jamming attacks and countermeasures should be analyzed in wireless sensor networks. Rather than acting as a secondary performance indicator, energy emerges as a governing factor that constrains attacker persistence, shapes defensive responsiveness, and strongly influences long-term network sustainability under adversarial interference.

By synthesizing two decades of research through an energy-centric lens, the analysis identifies consistent behavioral patterns across jamming strategies and indicates that defensive effectiveness is inherently coupled to energy overhead and protocol assumptions reported in the literature. These observations suggest that resilience against jamming cannot be assessed in isolation from energy dynamics, but should instead be understood as a balance between disruption, mitigation, and resource depletion.

Finally, the persistence of heterogeneous evaluation practices and simplified experimental assumptions highlights the need for greater methodological alignment in future research. Advancing toward energy-intelligent and deployment-relevant WSN designs will benefit from unified energy-performance evaluation, increased reliance on empirical validation, and adaptive mechanisms capable of responding proportionally to adversarial conditions.

### Future Research Directions

Future research should move toward the formalization of an energy–performance benchmarking framework that unifies attacker–defender energy modeling with standardized evaluation metrics. Such a framework should incorporate real-world empirical measurements, controlled interference scenarios, and cross-layer resilience indicators to enable reproducible and comparable assessments across studies. Furthermore, advancing the field will require the integration of testbed-based experimentation with modern WSN hardware, together with publicly available datasets that characterize coupled attacker–defender energy dynamics under jamming. Complementary research on energy-harvesting capabilities and adaptive learning mechanisms may enhance network endurance in hostile environments. Collectively, these directions can help establish a more standardized and deployment-relevant foundation for evaluating jamming resilience in energy-constrained WSNs.

## Figures and Tables

**Table 1:** Comparative summary of representative works on energy-aware jamming and anti-jamming strategies in WSNs.

Reference	Attack Type	Affected Layer(s)	Technique/Protocol	Energy Metric	Key Findings	Limitations
[22]	Random jamming	PHY/MAC	Probabilistic intermittent jamming model	Jammer and node energy usage	Random jamming reduces energy consumption by 5–10% compared to deceptive jamming due to periodic idle states.	Small-scale scenarios; no defensive analysis.
[23]	Constant jamming	PHY/MAC	IEEE 802.15.4 with XBee S1 nodes	Total consumption (J)	Constant jammer consumes 40% more energy than transmitter nodes and up to 118% more than reactive jammers.	No evaluation of defensive strategies.
[21]	Link-layer energy-efficient jamming	MAC	PSJ, PCJ, LPLJ	Residual energy	LMAC shows highest resilience under energy-efficient jamming.	Simulated topology only.
[28]	Energy-efficient jamming	MAC	S-MAC, LMAC, B-MAC variants	Energy per slot	Randomized LMAC is most robust.	No mobility analysis.
[29]	Jamming DoS	MAC	IEEE 802.15.4, T-MAC, S-MAC	Throughput/energy	T-MAC performs best under attack.	ZigBee-only simulations.
[30]	EDA	MAC (LoRaWAN)	LADE	Energy overhead	Detects EDAs with 0.3% overhead.	No PHY analysis.
[31]	General jamming	Multichannel MAC	EM-MAC	Duty cycle	Duty cycle kept at 5–7%.	No strong jamming tests.
[33]	Interference/jamming	Routing	HEERPOP	Total energy/lifetime	Improves lifetime vs. LEACH/OSEAP.	No targeted jamming tests.
[32]	Predictive jamming	Routing	JAM + LEACH	Energy per cluster	Lifetime improves ×3.	Limited scaling.
[35]	ML reactive jamming	PHY/MAC	LearJam	Energy per attack	400% more successful attacks.	No ML defenses.
[36]	PSJ jamming	MAC	PSJ	Jammer lifetime	Efficient long-lived jammer.	Requires periodic MAC.
[37]	Cooperative jamming	PHY/EH	RF harvesting	Harvested energy	Improves communication + reduces energy.	RF harvesting only.
[38]	Interference/eavesdropping	PHY/Industrial	SWIPT-like wireless energy transfer	RF power density	Enhances industrial WSN security through joint energy and information transfer.	Industrial-only applicability.
[39]	Full-duplex eavesdropper	PHY	Solar + RF energy harvesting	Harvested energy (mJ)	≈2 mJ harvested per interval; RF efficiency 50–75%.	Highly dependent on ambient conditions.
[40]	Friendly jamming	PHY/EH	RFID battery-free nodes	mJ harvested vs. distance	2.606 mJ at 1 m; 1.0749 mJ at 4 m enabling localization and battery-free operation.	Limited operating range.
[41]	Jamming + DoS detection	Link layer	Fuzzy logic IDS	Computational energy	Detects 75 anomalies vs. 45 using entropy with lower false positives.	Requires manual tuning.
[42]	Selective jamming	MAC (802.15.4)	Beacon/active-period jamming	Association loss/time	Critical nodes disconnected in 88 s under targeted jamming.	No defensive methods proposed.
[43]	Constant jamming DoS	Routing	AODV vs. DSR	Packet Sending Ratio (PSR)	CHs drop to PSR = 0.62 while other nodes remain near 1.0.	Does not evaluate proactive routing.
[44]	Selective low-energy jamming	MAC (TSCH)	WirelessHART route cracking	Listening time (ms)	Reconstructs routes in only 1920 ms on a 50-node testbed.	Limited to WirelessHART networks.
[45]	Sybil-related interference	Routing/PHY	Beamforming + reputation	SINR	90% of traffic converges to nodes with SINR > 4 dB.	Does not consider high-power jamming.
[46]	Selective jamming	TDMA/Routing	JAMMY	Packet loss (%)	Reduces packet loss to ≈4% (theoretical 3.3%).	Slot-management complexity.
[47]	Adaptive anti-jamming	Cross-layer	MDP + RS coding + power adjustment	Energy vs. PDR	Improves PDR > 50% and reduces energy ≈20%.	Requires precise channel modeling.
[27]	Constant jamming	Routing/Clustering	FCPA	Residual energy (%)	Maintains ≈63% energy and improves throughput >50%.	CH nodes deplete earlier.
[48]	DSSS anti-jamming	PHY	Enhanced DSSS	Break probability	Break probability reduced to 0.16 (1 jammer) and 0.28 (10 jammers).	Increased computational complexity.
[49]	Compressive sensing security	Application/PHY	CCPT	Current (A)/Tx energy	Consumes ≈0.08 A vs. ≈0.3 A (crypto), reducing Tx energy by 1.5–2.2×.	Not designed for reactive jamming.
[50]	Jammer localization	PHY/EH	RFID EH nodes	Harvested mJ/localization error	Mean error ≈4.5 m; supports battery-free operation.	Accuracy decreases under shadowing.
[51]	Jamming detection	ML/PHY	CNN Quantum Leap	Accuracy (%)	Achieves 95.4% accuracy on WSN-DS.	Dataset-dependent; no energy study.
[52]	Jamming DoS	PHY	Residual energy + RSSI + PDR	Throughput	Outperforms PDRSS under up to 5 attackers.	Limited-size simulation.
[53]	Reactive jamming detection	Hybrid	Two-phase detection	Detection time (s)	Detects jamming in <0.5 s with zero false positives.	Evaluated only on TelosB.
[54]	DoS/jamming	Routing/ML	DRBN	Accuracy/FPR/energy	98% accuracy and 2.8% FPR with reduced energy.	Scalability uncertain.
[55]	Selective forwarding	Network/PHY	E-Watchdog	Detection accuracy	Reduces false positives by 25% and increases accuracy by 10%.	Mobility reduces accuracy.
[56]	Game-theoretic jamming	Cross-layer	SPRT + Nash equilibrium	Energy ratio (E/P)	Identifies 16 equilibria balancing energy and security.	High modeling complexity.

**Table 2:** Protocol definition.

Parameter	Description
Main Question	Which jamming attacks are the most effective in WSNs, and what are the most energy-efficient strategies to mitigate them from an energy-aware perspective? Furthermore, how can the implementation of such countermeasures contribute to prolonging the overall network lifetime?
Aim	To identify the most effective jamming attacks targeting WSNs and to analyze energy-efficient mitigation strategies from an energy-aware perspective, with the aim of understanding how such countermeasures can contribute to extending the overall lifetime of the network.
Objective of Systematic Literature Review	The objective of this systematic literature review is to identify and synthesize previous research in the field of WSNs, focusing on the energy impact of jamming on both sensor nodes and the jammer itself. Additionally, the review aims to identify the most energy-efficient jamming and anti-jamming strategies reported in the literature.
Inclusion Criteria	The criteria for inclusion were: 1. Research examining the mechanisms and implications of jamming attacks within conventional WSNs. 2. Investigations categorizing jamming attacks, distinguishing between intentional (malicious) and non-intentional (accidental) interference. 3. Studies assessing the energy efficiency of jammers, exploring strategies to optimize power consumption while executing jamming operations. 4. Research focusing on energy harvesting, energy storage, and energy consumption dynamics in nodes subjected to jamming attacks. 5. Peer-reviewed journal articles, conference papers, and review articles indexed in Scopus and related to jamming, anti-jamming, or energy in WSNs.
Exclusion Criteria	The criteria for exclusion were: 1. Studies not written in English. 2. Studies published outside the 2004–2024 window. 3. Non-peer-reviewed sources (books, theses, white papers, technical reports, preprints).

**Table 3:** Energy-related performance metrics commonly reported in IEEE 802.15.4-based jamming and anti-jamming studies.

Metric	Definition	Typical Unit	Layer(s)	Role in Energy-Aware Jamming Analysis
Energy per delivered packet/bit	Average energy consumed by a node to successfully deliver one packet or bit under interference conditions	J/packet, J/bit	PHY/MAC	Primary indicator of energy efficiency degradation induced by jamming; captures the combined effects of retransmissions, channel sensing, and transmission overhead.
Radio-on time	Cumulative duration during which the radio remains in transmit, receive, or idle listening states	s, % of time	PHY/MAC	Reflects energy drain caused by prolonged channel sensing, reactive jamming, and repeated access attempts.
Duty cycle	Fraction of time a node operates in active versus sleep mode	%	MAC	Indicates deviations from low-power operation assumptions due to jamming-induced activity.
Average current consumption	Mean electrical current drawn by a node during node operation	mA	PHY	Hardware-oriented proxy for instantaneous energy stress under interference conditions.
Node or network lifetime	Elapsed time until battery depletion, first-node failure, or network partition, as explicitly defined by the study	h, days	Cross-layer	High-level indicator of long-term energy impact and operational sustainability under sustained jamming.
Retransmission count	Average number of retransmissions required per packet	packets	MAC	Indirect energy proxy linking jamming intensity to protocol-level overhead and energy waste.
Packet delivery ratio (PDR)	Ratio of successfully received packets to transmitted packets	%	MAC/Network	Availability-oriented metric frequently paired with energy indicators to contextualize performance degradation.
Attacker energy consumption	Energy expended by the jammer to disrupt legitimate communications	J, J/s	PHY	Enables attacker–defender energy asymmetry analysis when jointly reported in the evaluation.

**Table 4:** Common experimental assumptions reported in IEEE 802.15.4-based energy-aware jamming and anti-jamming studies.

Assumption Category	Typical Assumptions in the Literature	Impact on Energy and Jamming Evaluation
Channel model	AWGN, Rayleigh fading, or log-distance path-loss models; interference commonly abstracted as additive noise or packet collision probability	Channel abstraction directly affects retransmission rates, carrier sensing behavior, and estimated energy per delivered packet; simplified models may underestimate energy drain under reactive jamming.
Operating frequency band	2.4 GHz ISM and sub-GHz 868/915 MHz bands, often analyzed independently	Propagation characteristics and interference density differ significantly across bands, influencing jamming range, duty-cycle impact, and defender energy consumption.
Node density and topology	Sparse to moderate node densities (tens to hundreds of nodes); star-based or multi-hop tree/mesh topologies	Topology determines traffic concentration and retransmission cascades, shaping both local and network-wide energy depletion patterns.
Traffic model	Periodic sensing traffic with fixed packet sizes and low-to-moderate reporting rates	Traffic regularity facilitates timing-aligned or reactive jamming, amplifying energy inefficiency in duty-cycled protocols.
MAC configuration	CSMA/CA-based IEEE 802.15.4 MAC; fixed or adaptive duty cycling; bounded retransmission limits	MAC parameters govern idle listening, backoff behavior, and retransmission overhead, tightly coupling jamming intensity to energy consumption.
Jammer behavior and power	Constant, random, reactive, or selective jammers; jammer transmission power typically higher than that of sensor nodes	Jammer duty cycle and power level shape attacker–defender energy asymmetry and determine whether energy depletion or throughput loss dominates.
Evaluation platform	Simulation tools (NS-2, NS-3, OMNeT++); limited testbed experiments using TelosB, MicaZ, or CC1350 nodes	Simulation-only evaluations may overlook hardware-specific power states and timing effects, reducing the realism of reported energy metrics.
Energy model	Battery-based accounting with linear discharge assumptions; energy harvesting modeled as constant or stochastic input	Simplified energy models improve tractability but may obscure nonlinear battery behavior or harvesting variability in real deployments.

## Data Availability

All data supporting the findings of this study are contained within the article itself. No additional datasets were generated or analyzed outside the material presented in this manuscript. Figures, tables, and analytical results have been derived directly from the systematic review process and are fully documented herein.
